# Jasmonate Modulates Strawberry Susceptibility to Anthracnose by Activating SnRK2.1 to Regulate the WRKY50‐JAZ5 Module

**DOI:** 10.1111/pbi.70492

**Published:** 2025-12-12

**Authors:** Chuang Liu, Zhen Liu, Xia Li, Yating Chen, Ronghui Sun, Peijie Li, Qianqian Feng, Yuanhua Wang, Jie Ren, Qian Li, Bingbing Li

**Affiliations:** ^1^ Department of Pomology, College of Horticulture China Agricultural University Beijing China; ^2^ Jiangsu Vocational College of Agriculture and Forestry Jurong China; ^3^ Engineering and Technical Center for Modern Horticulture Jurong China; ^4^ Tobacco Research Institute of Chinese Academy of Agricultural Sciences Qingdao China

**Keywords:** JAZ5, MeJA, SnRK2.1, strawberry anthracnose, WRKY50

## Abstract

*Colletotrichum* spp., hemibiotrophic fungal pathogens, threaten global strawberry production. Jasmonate (JA) regulates plant‐*Colletotrichum* interactions, but its mechanisms remain unclear. Here we demonstrate that both exogenous methyl jasmonate (MeJA) treatment and elevated endogenous MeJA levels increase strawberry susceptibility to anthracnose. Two key JA biosynthesis genes, *FveAOS2* and *FveAOC3*, were identified as contributors to *Colletotrichum*‐induced susceptibility. Further analysis revealed that the FveSnRK2.1–FveWRKY50 phosphorylation module functions as an important molecular switch in regulating disease susceptibility. Specifically, *Colletotrichum* infection or MeJA application activates FveSnRK2.1, which phosphorylates FveWRKY50 at serine residue 88 (S88). This phosphorylation enhances the stability and transcriptional activity of FveWRKY50, leading to increased expression of *FveAOS2* and *FveAOC3*, higher MeJA accumulation and enhanced susceptibility. Notably, the strawberry JASMONATE‐ZIM DOMAIN (JAZ) protein FveJAZ5 suppresses susceptibility by directly interacting with FveWRKY50, thereby preventing its interaction with FveSnRK2.1 and inhibiting the activation of *FveAOS2* and *FveAOC3*. Upon pathogen attack or MeJA signalling, FveJAZ5 is degraded, thereby releasing FveWRKY50 from suppression. The study elucidates a *Colletotrichum*‐induced ‘JA signaling – JA biosynthesis’ positive feedback loop that drives strawberry susceptibility. Knocking out *FveWRKY50* and overexpressing *FveJAZ5* generated anthracnose‐resistant germplasms. These findings deepen understanding of plant‐*Colletotrichum* interactions and provide genes for resistant strawberry breeding.

## 1 Introduction

Anthracnose, caused by *Colletotrichum* spp., is one of the most destructive fungal diseases in agriculture, affecting over 760 plant species, including staple crops like maize, rice and soybean, as well as high‐value crops such as tea, coffee, tomato, chilli, mango and strawberry (Talhinhas and Baroncelli 2023). Anthracnose typically causes strawberry yield losses of 20%–40% under moderate conditions (Xie et al. 2010; Chung et al. 2020; Climate Risk Institute 2023), but losses can exceed 50% when favourable climatic conditions promote disease spread (Xie et al. 2010; Chung et al. 2020; Climate Risk Institute 2023). With climate change increasing global temperatures and humidity fluctuations, outbreaks can result in disease incidence exceeding 80%, especially during the nursery and early planting stages (Xie et al. 2010; Chung et al. 2020; Climate Risk Institute 2023). Traditional control methods, such as fungicides and crop rotation, are limited by pathogen resilience and fungicide resistance. This highlights the need for innovative solutions, such as molecular breeding and studies of pathogen‐host interactions. However, the molecular mechanisms underlying anthracnose pathogenesis remain largely uncharacterized.

Strawberry (*Fragaria × ananassa* Duch.) is a globally important horticultural crop with significant nutritional, health, and economic value. Wild strawberry ( *Fragaria vesca* L.), which has a diploid genome facilitating genetic manipulation, has become an essential research model for molecular and functional gene studies (Xing et al. 2020; Mao et al. 2022; Chen et al. 2023). To date, 23 *Colletotrichum* species have been identified as causal agents of strawberry anthracnose, with *C. fragariae* , *C. acutatum* , and *C. gloeosporioides* being the most prevalent pathogens (Ji et al. 2022). These pathogens exhibit a hemibiotrophic lifestyle, characterised by an initial biotrophic phase followed by necrotrophic expansion, enabling efficient evasion of host defences and successful colonisation of tissues. However, the complexity of hemibiotrophic infection mechanisms has limited our understanding of their regulatory networks (Chen et al. 2020; Zhang et al. 2020; Ji et al. 2022). While salicylic acid (SA)‐mediated pathways regulate defences against biotrophic pathogens, jasmonate (JA) signalling plays a central role in resistance to necrotrophs (Cao et al. 2025; Roychowdhury et al. 2025; Verma et al. 2025). Nevertheless, JA signalling can paradoxically enhance susceptibility to biotrophic pathogens, leaving its precise role in interactions with hemibiotrophs incompletely understood (Cao et al. 2025; Roychowdhury et al. 2025; Verma et al. 2025).

Notably, prior research has demonstrated that *Colletotrichum* infection dynamically modulates JA biosynthesis in strawberries and other plants, with contrasting effects depending on the plant species: increased JA biosynthesis correlates with heightened susceptibility in tea, maize and strawberry (Amil‐Ruiz et al. 2016; Gorman et al. 2020; Fang et al. 2021; Huang et al. 2023; Jeyaraj et al. 2023; Ma et al. 2023; Lv et al. 2024), whereas it induces enhanced resistance in papaya, grape and soybean (Kuralarasi et al. 2018; Zhu et al. 2022; Wang et al. 2024; Yang et al. 2024; Zhang et al. 2024). Consistent with this, anthracnose‐induced JA accumulation is well documented in several strawberry cultivars (Amil‐Ruiz et al. 2016; Fang et al. 2021; Ma et al. 2023). A recent study revealed that the application of methyl jasmonate (MeJA) increased MeJA content and susceptibility to anthracnose in ‘Sweet Charlie’ strawberries, while the application of SA increased its resistance to anthracnose, and this responsiveness was associated with HSP17.4 protein (Fang et al. 2021). Another study found that overexpression of the mannose‐binding lectin gene *MANNOSE‐BINDING LECTIN 1* (*MBL1*) decreased JA content and increased the resistance to anthracnose in ‘Sveva’ strawberries (Ma et al. 2023). While previous studies have highlighted the importance of JA in modulating plant response to anthracnose, the exact molecular pathways and regulatory networks remain unclear. Recent findings in tea revealed that the CsJAZ1‐CsMYC2.2 module regulates *CsGSTU45* gene expression and protein levels to regulate JA‐mediated susceptibility to *C. camelliae* in tea plants (Lv et al. 2024). Another study found that CpWRKY50 directly binds to W‐box motifs in the promoters of two JA signalling‐related genes, *CpMYC2* and *CpPR4*, activating their expression and positively regulating anthracnose resistance in papaya through the promotion of JA signalling (Wang et al. 2024). However, the regulatory networks connecting JA biosynthesis, signal transduction, and downstream effectors in plant‐*Colletotrichum* interactions are still poorly characterised. Additionally, given that previous studies have largely relied on transient or heterologous expression systems in model plants such as *Arabidopsis*, the feasibility and specific genes suitable for genome‐editing approaches to generate resistant germplasm require further validation.

Compared to the limited understanding of JA in anthracnose resistance, key components involved in JA biosynthesis and signalling transduction have been well established (Chen et al. 2012; Zhai et al. 2020; An et al. 2022; Cao et al. 2025; Li et al. 2025; Roychowdhury et al. 2025; Verma et al. 2025; Wu et al. 2025). JA biosynthesis begins with α‐linolenic acid oxidation and proceeds through a conserved enzymatic cascade involving lipoxygenases (LOXs), allene oxide synthase (AOS), allene oxide cyclase (AOC) and OPDA reductase 3 (OPR3). Notably, AOS and AOC catalyse the committed steps by converting 13‐hydroperoxyoctadecatrienoic acid (13‐HPOT) to 12‐oxophytodienoic acid (12‐OPDA), which determines metabolic flux toward JA production. The bioactive conjugate jasmonoyl‐isoleucine (JA‐Ile) accumulates at low levels under normal growth conditions, where JASMONATE‐ZIM DOMAIN (JAZ) repressors inhibit transcription factors such as MYC2, maintaining signalling in an inactive state. Upon pathogen attack or other stress conditions, rapid JA‐Ile biosynthesis triggers CORONATINE INSENSITIVE1 (COI1)‐mediated ubiquitination and subsequent degradation of JAZ proteins via the 26S proteasome. This derepression allows MYC2 and other transcription factors to activate stress responsive genes. Crucially, JA's pleiotropic effects require precise regulatory mechanisms to balance stress responses with plant growth and development (Cao et al. 2025; Roychowdhury et al. 2025; Verma et al. 2025). Dissecting pathogen‐specific response modules from developmental signalling cascades is associated with constitutive defence activation. The JAZ protein family is central to this regulatory balance, leveraging its structural diversity, functional redundancy, spatiotemporal expression specificity, hierarchical post‐translational modifications and complex interaction networks—particularly through interactions with transcription factors—to achieve precise regulation of distinct developmental and stress‐responsive signalling pathways (Garrido‐Bigotes et al. 2018; Cao et al. 2025; Roychowdhury et al. 2025; Verma et al. 2025). However, the role of JAZ proteins in anthracnose resistance remains poorly understood, and the specific JAZ members and protein complexes modulating this process in strawberry had not been identified. Thus, elucidating the key genes involved in JA biosynthesis and signalling is essential to advance our understanding of the regulatory mechanisms underlying anthracnose resistance.

Anthracnose pathogens activate both Pattern‐Triggered Immunity (PTI) and Effector‐Triggered Immunity (ETI) in host plants (Jiang et al. 2022; Zhou et al. 2023). This initial recognition triggers conserved downstream signalling events, including reactive oxygen species (ROS) bursts, cellular Ca^2+^ influxes, reversible protein phosphorylation and transcriptional reprogramming (Jiang et al. 2022; Zhou et al. 2023). To unravel the complexity of plant defences against *Colletotrichum* spp., research has extended beyond characterising the roles of SA and JA in anthracnose resistance. Transcriptomic, metabolomic and other multi‐omics approaches are now widely employed to identify key regulatory genes and underlying mechanisms. These omics analyses consistently highlight the central importance of specific transcription factor (TF) and protein kinase (PK) families in mediating plant responses to anthracnose infection. WRKY and NAC (NAM, ATAF1/2 and CUC2) TFs, alongside RLK (receptor‐like kinase) and MAPK (mitogen‐activated protein kinase) PKs, are recurrently implicated in resistance across diverse species (Fang et al. 2012; Wang et al. 2017; Salinas et al. 2019; Chandra et al. 2021; Shan et al. 2021; Adhikari et al. 2022; Zou et al. 2022, 2025; Mu et al. 2024; Cao et al. 2025; Chen et al. 2025; Ma et al. 2025; Roychowdhury et al. 2025; Verma et al. 2025). Among PKs, SnRK2 (Sucrose non‐fermenting 1‐related protein kinase 2) family members are well‐established core regulators of abiotic stress signalling, but their roles in plant immunity are emerging and less defined (Lee et al. 2015; Mao et al. 2019; Mondal et al. 2024), particularly in response to hemibiotrophic pathogens like *Colletotrichum. WRKY* transcription factors, a plant‐specific gene family, are well‐documented as key players in immune responses alongside growth and development (Chen et al. 2025). Studies across species indicate that different WRKY homologues can play distinct, sometimes opposing, roles in regulating anthracnose resistance (Mu et al. 2024; Chen et al. 2025). However, functional knowledge of WRKYs in strawberry, especially regarding jasmonic acid (JA)‐mediated defence, remains limited. Notably, phosphorylation cascades involving MAPK‐WRKY modules have been shown to contribute to plant defence in tobacco and apple (Ishihama et al. 2011; Shan et al. 2021). Similarly, transient expression assays in strawberry fruit have implicated FaWRKY1, FaRLK10 and the small peptide FaRALF33 in regulating anthracnose outcomes (Encinas‐Villarejo et al. 2009; Higuera et al. 2019; Merino et al. 2019; Negrini et al. 2020; Han, Salinas, et al. 2025). Collectively, omics studies have generated a substantial inventory of candidate TFs and PKs potentially orchestrating defence against *Colletotrichum*. However, significant research gaps remain. While progress has been made in understanding SA‐mediated biotrophic and hemibiotrophic resistance, the signalling mechanisms underpinning JA‐mediated hemibiotrophic susceptibility or resistance—including the specific TFs and PKs involved—are largely unexplored (Jeyaraj et al. 2023; Cao et al. 2025; Roychowdhury et al. 2025; Verma et al. 2025). Despite generating extensive candidate regulators, functional validation lags significantly, particularly regarding JA‐mediated resistance mechanisms and novel PK classes beyond RLKs and MAPKs. This delay is primarily attributed to challenges such as the functional redundancy and complexity within large gene families (e.g., WRKY, RLK, MAPK), coupled with technical hurdles in genetic manipulation, particularly in non‐model and perennial species.

In this study, we elucidate an important signalling pathway that governs JA‐mediated anthracnose susceptibility in strawberry. We identified the transcription factor FveWRKY50, the protein kinase FveSnRK2.1 and the JA‐signalling repressor FveJAZ5 as key regulators. This FveSnRK2.1‐FveWRKY50‐FveJAZ5 module finely tunes the ‘JA signaling – JA biosynthesis’ positive feedback loop, thereby determining anthracnose resistance outcomes. Overexpression of *FveJAZ5* effectively dampens excessive JA signalling, while targeted knockout of *FveWRKY50* disrupts the pathogenic feedback loop. Our work reveals a previously unrecognised signalling mechanism underlying JA‐mediated anthracnose resistance and identifies potential candidates for breeding resistant cultivars and advancing molecular diagnostics in strawberry cultivation.

## 2 Results

### 2.1 FveWRKY50 Positively Regulates the Susceptibility of Strawberry to Anthracnose

**FIGURE 1:**
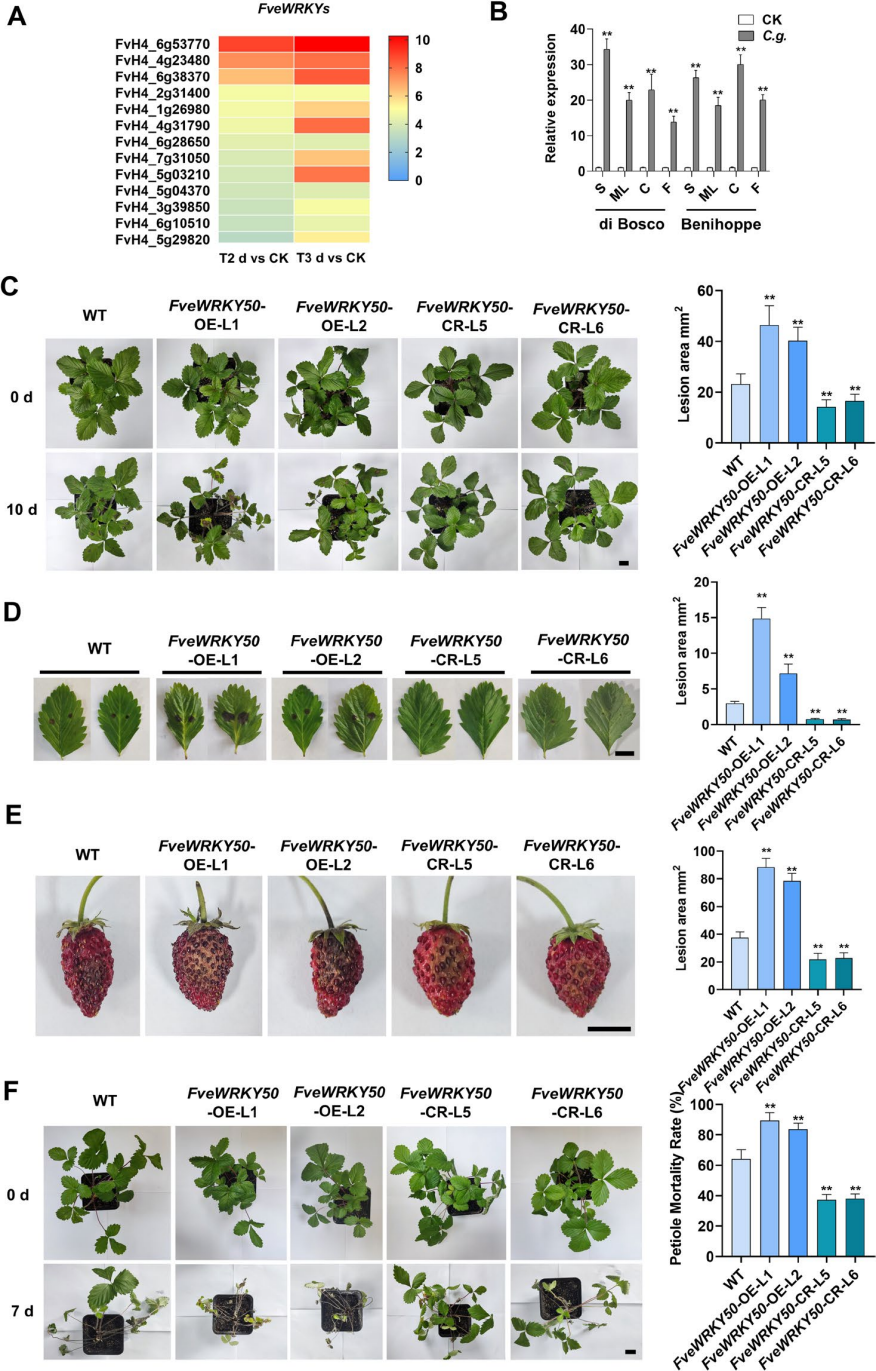
FveWRKY50 positively regulates strawberry susceptibility to anthracnose across multiple organs. (A) Expression profiles of *FveWRKY* family members in *C. gloeosporioides*‐infected ‘di Bosco’ strawberry seedlings at 2 and 3 days post‐inoculation (dpi), as determined by RNA‐seq analysis. (B) Expression levels of *FveWRKY50* in *C. gloeosporioides*‐infected ‘di Bosco’ and ‘Benihoppe’ strawberries, as measured by qRT‐PCR. S, seedlings; ML, mature leaves; C, crowns; F, fruits; *C.g*. *Colletotrichum gloeosporioides*. Values are means ± standard error of mean (SEM) of three biological replicates. Statistical significance was determined by Student's *t* test (*p < 0.05, **p < 0.01). (C–E) Phenotypic analysis and lesion area quantification in (C) attached leaves (droplet‐inoculated, 10 dpi), (D) detached leaves (droplet‐inoculated, 5 dpi), and (E) fruits (droplet‐inoculated, 7 dpi) of WT, *FveWRKY50*‐ OE, and *FveWRKY50*‐CR strawberry plants. Values are means ±SEM of three biological replicates (10 samples/replicate). Statistical significance was determined by Student's *t* test (*p < 0.05, **p < 0.01). (C) Scale bars, 2 cm; (D, E) Scale bars, 1 cm. (F) Phenotypic analysis and petiole mortality rate quantification in WT, *FveWRKY50*‐ OE and *FveWRKY50*‐CR strawberry plants following crown infection at 7 dpi. Values are means ±SEM of three biological replicates (10 samples/replicate). Statistical significance was determined by Student's *t* test (*p < 0.05, **p < 0.01). Scale bars, 2 cm.

To investigate the regulatory mechanisms underlying anthracnose resistance in strawberry, we conducted RNA‐seq analysis on *C. fructicola* infected ‘di Bosco’ strawberry seedlings to identify key regulatory genes, with a particular focus on transcription factors. As shown in Figure S1A and Data S1–S3, the WRKY family exhibited the most significant transcriptional changes among all transcription factor families. Notably, *FveWRKY50* showed the highest upregulation levels at both 2 and 3 days post‐infection (dpi) compared to other differentially expressed *WRKY* genes (Figure 1A). Consistent with this finding, *FveWRKY50* expression was markedly induced in leaves, crowns and fruits of both diploid ‘di Bosco’ and octoploid ‘Benihoppe’ upon anthracnose infection (Figure 1B), suggesting its conserved role in organ‐wide responses to anthracnose.

Subsequently, we generated Overexpression (OE) and CRISPR/Cas9‐mediated gene editing (CR) lines in strawberries to investigate the specific role of *FveWRKY50* in the defence response against anthracnose (Figure S1B–D). The results demonstrated that *FveWRKY50*‐OE lines exhibited significantly increased susceptibility to anthracnose in both attached and detached leaves as well as in fruits, whereas *FveWRKY50*‐CR mutants displayed markedly enhanced resistance to this disease (Figure 1C–E). Considering that anthracnose primarily causes the mortality of strawberry plants by infecting the crown tissue, we simulated this process by inoculating the crown region of strawberry plants (Figure S1E). Four days after anthracnose infection, the strawberry crown started to exhibit infection symptoms. Simultaneously, the leaves emerging from the crown area began to display infection symptoms and gradually wilted. By the eighth day post‐infection, the entire plant succumbed to the disease. Consistent with our previous findings in in vivo systems, overexpression of *FveWRKY50* significantly increased whole‐plant susceptibility to crown‐infected anthracnose, whereas the knockout mutant showed enhanced resistance to this infection (Figure 1F). These findings revealed that FveWRKY50 acts as a positive regulator of strawberry susceptibility to anthracnose, and its manipulation by CRISPR/Cas9‐mediated gene editing could potentially be a strategy for breeding anthracnose—resistant strawberry varieties.

We further analysed our RNA‐Seq data from anthracnose‐infected strawberries to identify upstream regulators of *FveWRKY50* expression. The transcription factor *FveMYB108* (*FvH4_5g11930*) was the most highly upregulated transcription factor, a finding validated by qRT‐PCR (Data S1–S3; Figure S2A). Functional studies showed that transient overexpression of *FveMYB108* compromised resistance to anthracnose in octoploid strawberry fruit and concurrently induced the expression of *FveWRKY50*, *FveAOS2* and *FveAOC3* (Figure S2B–D). This indicates that *FveMYB108* acts as a negative regulator of disease resistance upstream of *FveWRKY50*. However, EMSA results revealed that *FveMYB108* could not directly bind to probes containing MBS motifs in the *FveWRKY50* promoter (Figure S2E), suggesting an indirect mechanism underlying its transcriptional activation.

### 2.2 FvWRKY50 Regulates the Susceptibility of Strawberry to Anthracnose by Modulating JA Biosynthesis

**FIGURE 2:**
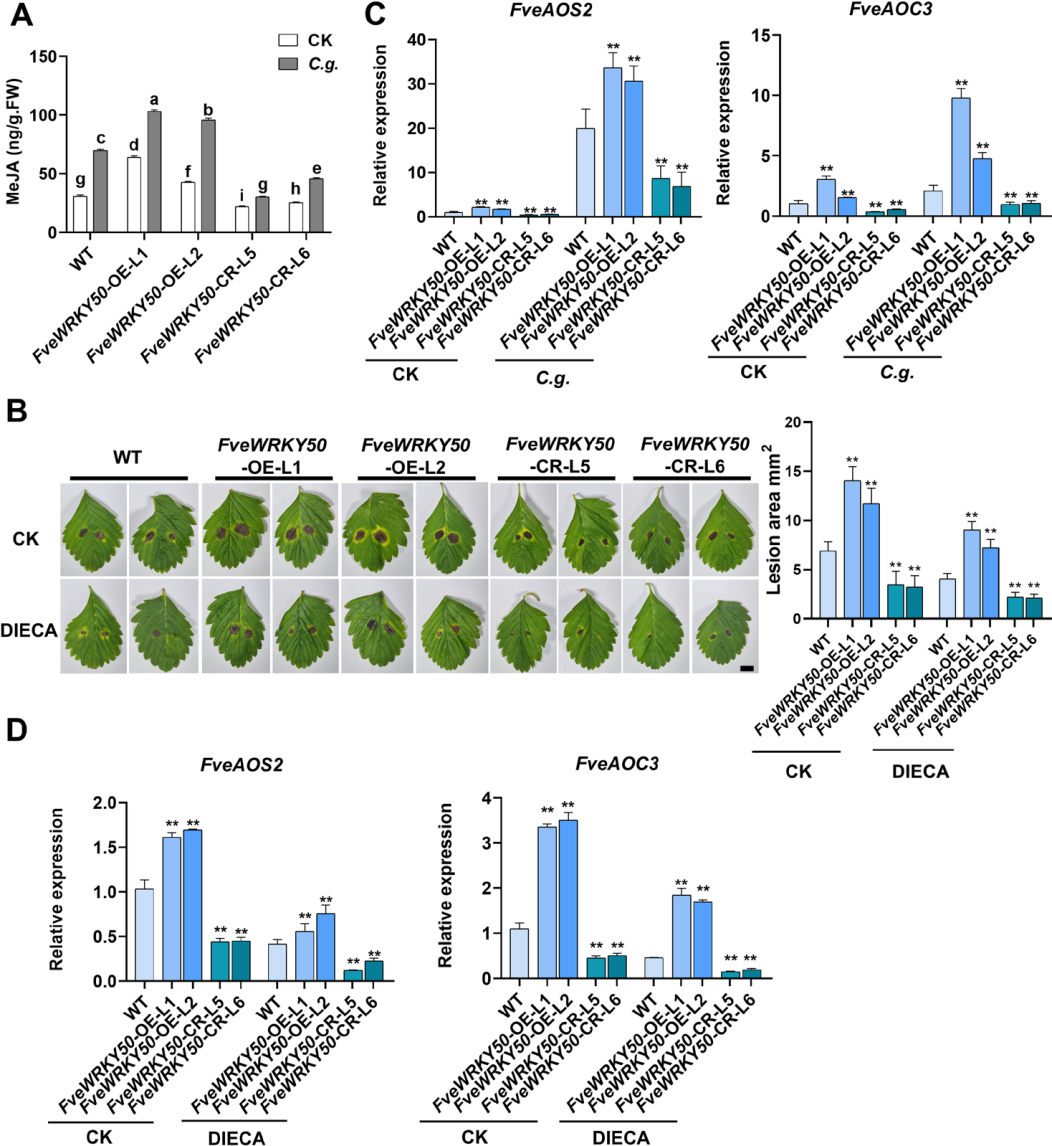
FveWRKY50 modulates JA biosynthesis to enhance strawberry susceptibility to anthracnose infection. (A) MeJA content in *C. gloeosporioides*‐infected ‘di Bosco’ strawberry leaves at 5 dpi. Values are means ±SEM of three biological replicates (each with 3 technical replicates). Different lowercase letters indicate significant differences (one‐way ANOVA, Tukey's test). (B) Phenotypic analysis and lesion area quantification in WT, *FveWRKY50*‐ OE, and *FveWRKY50*‐CR strawberry leaves pre‐treated with DIECA (50 μM, 24 h prior to anthracnose infection). Values are means ±SEM of three biological replicates (each with 10 technical replicates). Statistical significance was determined by Student's *t* test (*p < 0.05, **p < 0.01). (C) qRT‐PCR analysis of *FveAOS2* and *FveAOC3* expression in *C. gloeosporioides*‐infected ‘di Bosco’ leaves at 3 dpi. Values are means ±SEM of three biological replicates. Statistical significance was determined by Student's *t* test (*p < 0.05, **p < 0.01). (D) Expression levels of *FveAOS2* and *FveAOC3* in *C. gloeosporioides*‐infected ‘di Bosco’ leaves pre‐treated with DIECA (100 μM, 24 h prior to anthracnose infection) at 3 dpi. Values are means ±SEM of three biological replicates. Statistical significance was determined by Student's *t* test (*p < 0.05, **p < 0.01)

Previous studies have demonstrated the potential role of JA in modulating strawberry susceptibility to anthracnose defence (Fang et al. 2021; Ma et al. 2023). Therefore, we investigated hormonal changes and observed that, compared to wild‐type, the content of OPDA, JA, JA‐Ile and MeJA was all significantly upregulated in *FveWRKY50*‐OE leaves (Figure S3A and Figure 2A). To further confirm the conserved role of JA in strawberry anthracnose susceptibility, we detected MeJA changes in anthracnose‐infected diploid ‘di Bosco’ and octoploid ‘Benihoppe’. The results showed that anthracnose significantly induced an increase in MeJA content across different organs in both varieties, and pretreatment with the JA biosynthesis inhibitor diethyldithiocarbamic acid (DIECA) not only repressed the biosynthesis of MeJA but also decreased infection symptoms and reduced lesion area in strawberries (Figure S3B‐D). These findings demonstrate that anthracnose‐triggered endogenous JA biosynthesis is a conserved susceptibility determinant in diploid ‘di Bosco’ and octoploid ‘Benihoppe’. Notably, *FveWRKY50*‐OE strawberries exhibited a more pronounced increase in MeJA levels, whereas *FveWRKY50*‐CR strawberries showed a significant reduction (Figure 2A). Furthermore, pretreatment with DIECA effectively suppressed infection symptoms and reduced lesion areas (Figure 2B).

To identify the key biosynthesis genes involved in anthracnose‐induced JA biosynthesis, we analysed the expression patterns of the limiting enzyme‐coding genes *FveAOSs* and *FveAOCs*. A total of five *FveAOSs* and three *FveAOCs* were identified in strawberry (Figure S4). Although the expression patterns of these genes varied across different organs, *FveAOS2* and *FveAOC3* were consistently the most highly regulated genes in both diploid and octoploid strawberries, suggesting a conserved response pattern for these two genes during anthracnose infection (Figure S4). Notably, *FveAOS2* exhibited negligible basal expression in various tissues but showed > 200‐fold transcriptional induction upon anthracnose infection in both ‘di Bosco’ and octoploid ‘Benihoppe’ seedlings (Figure S4A and Data S4). Further analysis revealed that the time course of anthracnose‐induced JA accumulation was closely correlated with the upregulated expression levels of *FveAOS2* and *FveAOC3* (Figure S5A), and DIECA treatment could suppress the changes of *FveAOS2* and *FveAOC3* expression (Figure S5B). Consistently, transient overexpression of *FveAOC3* led to increased MeJA content but significantly reduced resistance to anthracnose infection (Figure S5C,D). Collectively, these data indicate that *FveAOS2* and *FveAOC3* serve as key regulatory genes involved in anthracnose‐induced JA biosynthesis.

In agreement with these findings, compared to wild‐type strawberries, anthracnose‐induced expression levels of *FveAOS2* and *FveAOC3* were significantly enhanced in *FveWRKY50*‐OE strawberries and markedly suppressed in *FveWRKY50*‐CR strawberries (Figure 2C). Furthermore, FveWRKY50 enhanced the repression effects of DIECA on anthracnose‐induced *FveAOS2* and *FveAOC3* expression levels (Figure 2D).

### 2.3 FveSnRK2.1 Phosphorylates FveWRKY50 at Serine 88

**FIGURE 3:**
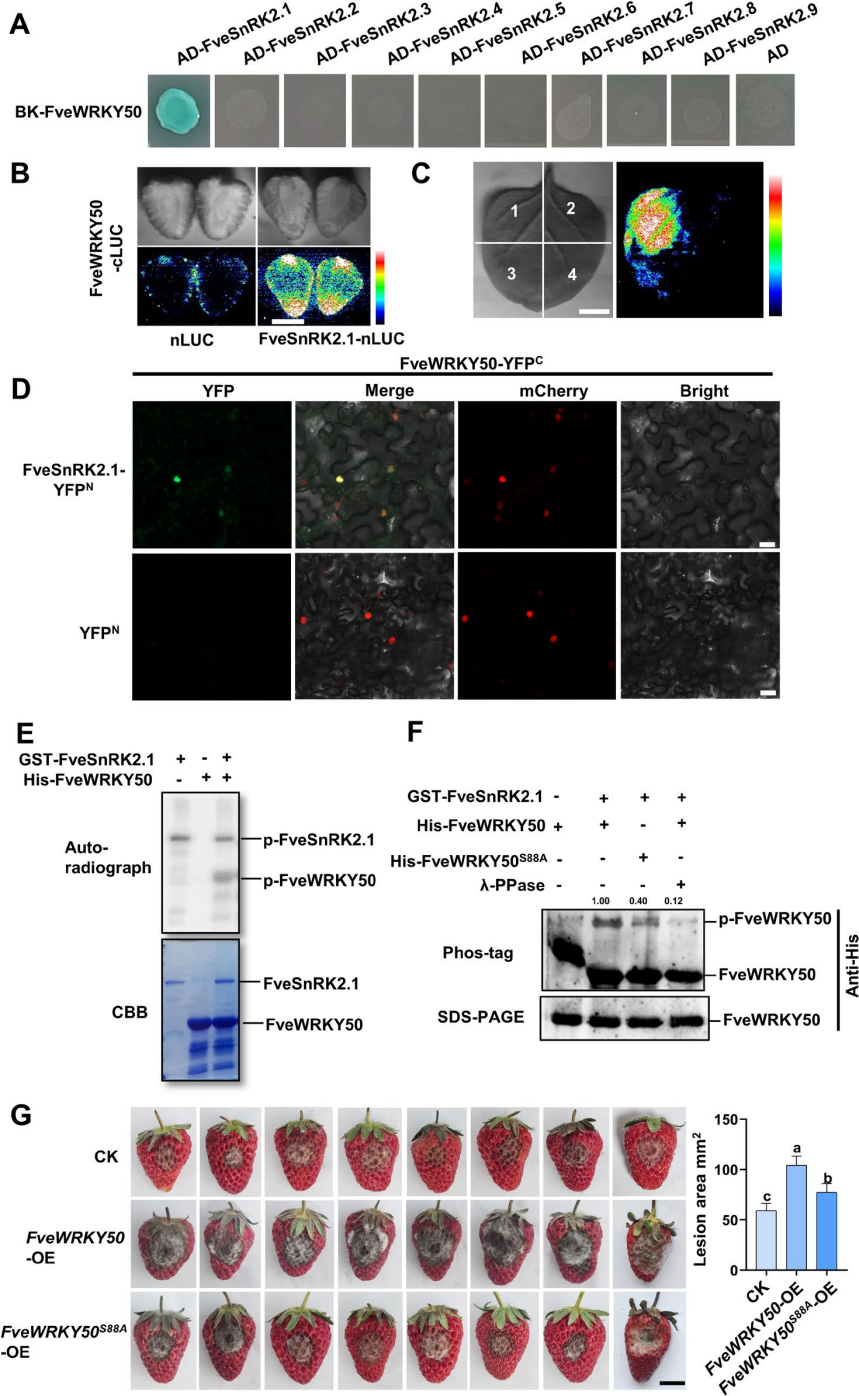
FveSnRK2.1 phosphorylates FveWRKY50 at Ser88. (A) Y2H assay for interaction between FveWRKY50 and FveSnRK2 family members. Y2H assays were conducted using pGBKT7 and pGADT7 vectors in *Saccharomyces cerevisiae* AH109. (B, C) LCI assay validated the interaction between FveWRKY50 and FveSnRK2.1 in strawberry fruits (B) and tobacco leaves (C). 1, FveWRKY50‐cLUC+FveSnRK2.1‐nLUC; 2, FveWRKY50‐cLUC+nLUC; 3, cLUC+ FveSnRK2.1‐nLUC; 4, cLUC+nLUC. Scale bars, 1 cm. (D) BiFC assay detected FveWRKY50 and FveSnRK2.1 interacted in nuclear of tobacco leaves. Scale bars, 20 μm. A nuclear marker fused with red fluorescence (pSuper::*NF‐YA4*‐mCherry) was used to visualise the cell nucleus. (E) In vitro kinase assay showing FveSnRK2.1‐mediated phosphorylation of FveWRKY50. The phosphorylated FveSnRK2.1 and FveWRKY50 proteins were visualised by autoradiography. Proteins were detected by western blot with anti‐GST and anti‐His antibodies for measuring GST‐FveSnRK2.1 and His‐FveWRKY50, respectively. CBB staining was used to detect the recombinant FveSnRK2.1 and FveWRKY50 proteins. (F) Phos‐tag assay confirming FveSnRK2.1‐mediated phosphorylation of FveWRKY50 at Ser88. His‐FveWRKY50^S88A^ was generated by mutating Ser88 to Ala in the recombinant protein. FveWRKY50 protein level and its phosphorylation status were detected using an anti‐His antibody (1:1000 dilution, CWBIO) as indicated. λ‐PPase treatment abolished the retarded mobility band, verifying that the band represented phosphorylated FveWRKY50. (G) Transient overexpression of *FveWRKY50* and its phosphorylation‐deficient mutant *FveWRKY50* ^*S88A*^ was performed to evaluate their roles in modulating susceptibility to anthracnose infection in octoploid ‘Benihoppe’ fruits. Phenotypic analysis and lesion area quantification in fruits were determined. CK, transient expression of empty vector in octoploid ‘Benihoppe’ fruits as control. Values are means ±SEM of three biological replicates (10 samples/replicate). Statistical significance was determined by Student's *t* test (*p < 0.05, **p < 0.01). Scale bars, 1 cm.

To investigate the upstream regulatory mechanism of FveWRKY50, especially the post‐transcriptional regulatory mechanism, we performed pull‐down coupled with mass spectrometry to screen potential interacting proteins. Notably, two members of the SnRK2 protein kinase family were identified, with FveSnRK2.1 showing the highest identification score among all candidate regulatory proteins (Data S5 and Figure S6A). To address potential functional redundancy within the SnRK2 family, we systematically tested interactions between FveWRKY50 and all nine annotated FveSnRK2 members using yeast two‐hybrid (Y2H) assays. Notably, FveWRKY50 specifically interacted with FveSnRK2.1 but showed no detectable binding to other family members under identical conditions (Figure 3A). The interaction was further validated *in planta* through luciferase complementation imaging (LCI) assays in *Nicotiana benthamiana* leaves and strawberry fruits (Figure 3B,C), while bimolecular fluorescence complementation (BiFC) assays confirmed nuclear localization of the interaction (Figure 3D).

To determine whether FveSnRK2.1 directly phosphorylates FveWRKY50, we conducted in vitro kinase assays using purified GST‐FveSnRK2.1 and His‐FveWRKY50 proteins. Immunoblotting with anti‐His antibody revealed robust phosphorylation of His‐FveWRKY50 by GST‐FveSnRK2.1 (Figure 3E). MS analysis pinpointed serine 88 (S88) as the primary phosphorylation site on FveWRKY50 mediated by FveSnRK2.1 (Figure S6B). The functional relevance of S88 was further corroborated by Phos‐tag assays, wherein mutation of S88 to alanine (S88A) significantly reduced FveSnRK2.1‐mediated phosphorylation (Figure 3F). To assess the importance of this phosphorylation site, we transiently overexpressed *FveWRKY50* and its phospho‐dead mutant *FveWRKY50* ^*S88A*^ in octoploid strawberry fruits. Compared to *FveWRKY50*‐OE fruits, those overexpressing *FveWRKY50* ^*S88A*^ showed significantly reduced susceptibility to anthracnose (Figure 3G). Although pathogen infection in the mutant fruits was not completely blocked relative to empty vector controls (Figure 3G), the results indicate that FveSnRK2.1‐mediated phosphorylation at S88 is important, but not the sole determinant, in regulating anthracnose resistance. Collectively, our data establish FveSnRK2.1 as a direct upstream kinase that phosphorylates FveWRKY50 at S88, and this post‐translational modification is important for FveWRKY50's function in disease resistance.

Since our previous work demonstrated that FveMAPK3 phosphorylates FveWRKY50 under low temperature to regulate anthocyanin biosynthesis (Chen et al. 2023), we investigated its potential role in anthracnose susceptibility. Due to the abnormal developmental phenotypes of *FveMAPK3* knockout mutants (Figure S7A), we used *FveMAPK3*‐overexpression (OE) lines for pathogen infection assays. However, no significant difference in susceptibility was observed between the *FveMAPK3*‐OE lines and the wild type (Figure S7B,C), suggesting that FveMAPK3 is not involved in regulating anthracnose resistance under normal conditions.

### 2.4 FveSnRK2.1 Positively Regulates Susceptibility to Anthracnose by Modulating FveWRKY50‐ *FveAOS2* / *FveAOC3* Module in Strawberries

**FIGURE 4:**
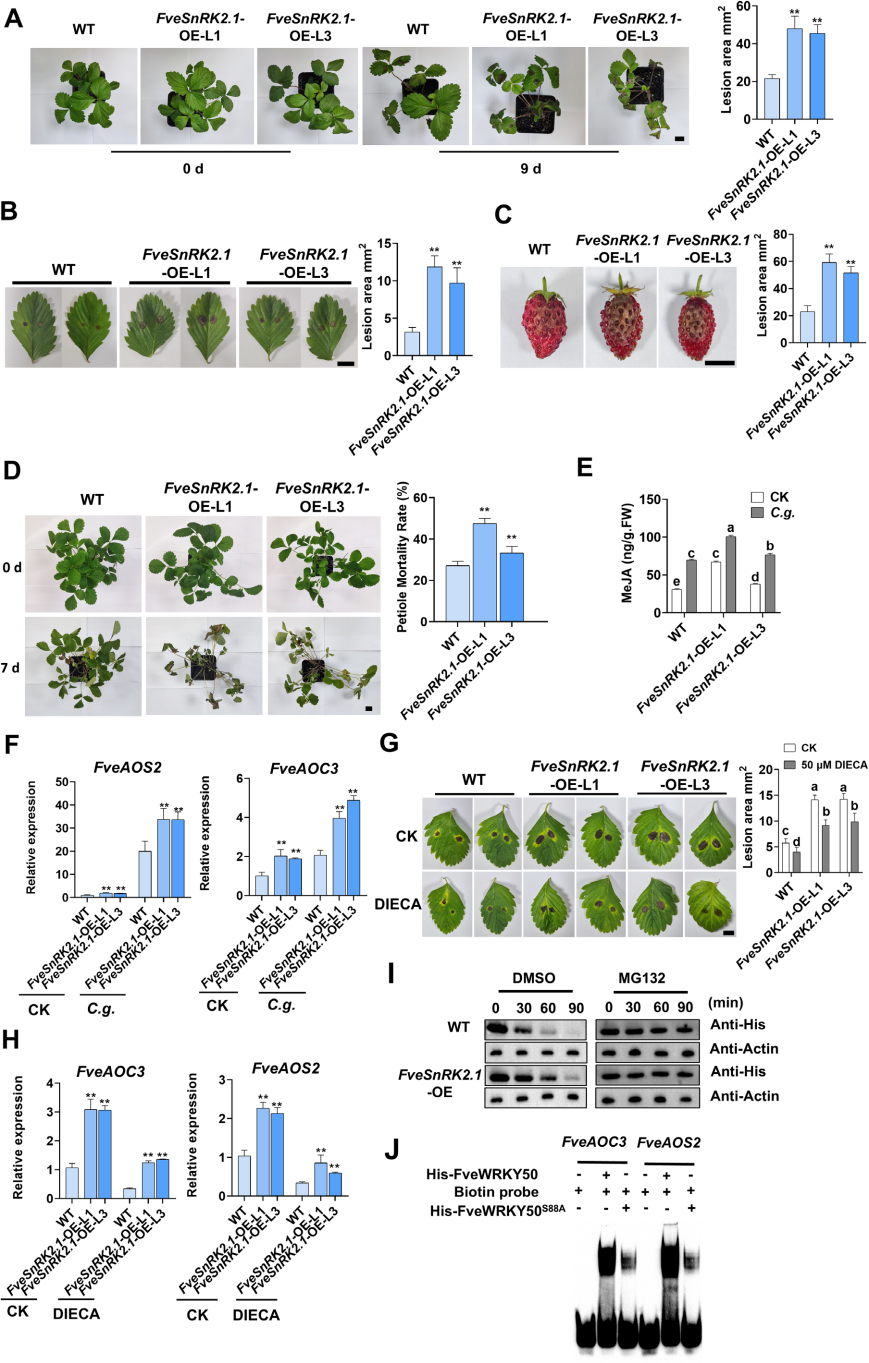
FveSnRK2.1 regulates strawberry susceptibility to anthracnose by modulating FveWRKY50 protein stability and transcriptional activity. (A–C) Phenotypic analysis and lesion area quantification in (A) attached leaves (droplet‐inoculated, 9 dpi), (B) detached leaves (droplet‐inoculated, 5 dpi), and (C) fruits (droplet‐inoculated, 7 dpi) of WT and *FveSnRK2.1*‐ OE plants. A, Scale bars, 2 cm; (B, C) Scale bars, 1 cm. Values are means ±SEM of three biological replicates (each with 10 technical replicates). Statistical significance was determined by Student's *t* test (*p < 0.05, **p < 0.01). (D) Phenotypic analysis and petiole mortality rate quantification of WT and *FveSnRK2.1*‐OE strawberry plants following crown infection at 7 dpi. Scale bars, 2 cm. Values are means ±SEM of three biological replicates (each with 10 technical replicates). Statistical significance was determined by Student's *t* test (*p < 0.05, **p < 0.01). (E) MeJA content in WT and *FveSnRK2.1*‐OE strawberry leaves infected with *C. gloeosporioides* at 3 dpi. Values are means ±SEM of three biological replicates. Different lowercase letters indicate significant differences (one‐way ANOVA, Tukey's test). (F) qRT‐PCR detecting *FveAOS2* and *FveAOC3* expression in WT and *FveSnRK2.1*‐OE strawberry leaves infected with *C. gloeosporioides* at 3 dpi. Values are means ±SEM of three biological replicates. Statistical significance was determined by Student's *t* test (*p < 0.05, **p < 0.01). (G) Phenotypic analysis and lesion area quantification in *C. gloeosporioides* ‐infected WT and *FveSnRK2.1*‐OE strawberry leaves pre‐treated with DIECA (50 μM, 24 h prior to anthracnose infection) at 5 dpi. Values are means ±SEM of three biological replicates (each with 10 technical replicates). Different lowercase letters indicate significant differences (one‐way ANOVA, Tukey's test). Scale Bar, 1 cm. (H) qRT‐PCR detecting *FveAOS2* and *FveAOC3* expression in *C. gloeosporioides*‐infected WT and *FveSnRK2.1*‐OE strawberry leaves pre‐treated with DIECA (50 μM, 24 h prior to anthracnose infection) at 3 dpi. Values are means ±SEM of three biological replicates. Statistical significance was determined by Student's *t* test (*p < 0.05, **p < 0.01). Scale Bar, 1 cm. (I) Cell‐free degradation assay showing FveSnRK2.1 enhanced His‐FveWRKY50 stability. Recombinant His‐FveWRKY50 proteins were incubated with total protein extracts from WT or *FveSnRK2.1*‐OE fruits in the presence of 50 μM MG132 (proteasome inhibitor) or DMSO followed by western blot with anti‐His antibody. (J) EMSA confirming that phosphor‐dead mutant of FveWRKY50 repressed its binding to the promoter of *FveAOS2* and *FveAOC3*. A phospho‐dead mutant His‐FveWRKY50^S88A^ was generated by mutating Ser88 to Ala.

To investigate the role of FveSnRK2.1 in strawberry anthracnose response, we generated *FveSnRK2.1*‐overexpressing (*FveSnRK2.1*‐OE) strawberry lines (Figure S6C). Phenotypic analysis revealed that *FveSnRK2.1*‐OE plants exhibited significantly increased susceptibility to anthracnose in leaves, fruits and whole plants with crown infection (Figure 4A–D). Compared to WT, anthracnose‐induced MeJA accumulation was more significantly enhanced in *FveSnRK2.1*‐OE strawberries (Figure 4E), which correlated with upregulated expression levels of *FveAOS2* and *FveAOC3* (Figure 4F). In line with these findings, DIECA application partially rescued the susceptibility of *FveSnRK2.1*‐OE strawberries and repressed the expression of *FveAOS2* and *FveAOC3*, thus indicating that FveSnRK2.1‐mediated susceptibility is associated with *FveAOS2*‐ and *FveAOC3*‐regulated endogenous MeJA content (Figure 4G,H).

Cell‐free protein degradation assays revealed that the degradation of His‐FveWRKY50 was significantly repressed in *FveSnRK2.1*‐OE protein extracts compared to WT, and this repression was abolished by the proteasome inhibitor MG132 (Figure 4I). These results indicate that FveSnRK2.1 positively regulates FveWRKY50 protein stability via the proteasome pathway.

To further explore whether FveSnRK2.1‐mediated phosphorylation of FveWRKY50 regulates the expression of *FveAOS2* and *FveAOC3*, we performed an EMSA assay. The results showed that FveWRKY50 bound to the W‐box elements in the promoters of *FveAOS2* and *FveAOC3*, and this binding affinity was significantly reduced in the presence of the phosphorylation‐deficient mutant FveWRKY50^S88A^ (Figure 4J). These results aligned with the altered anthracnose‐induced expression patterns of *FveAOS2* and *FveAOC3* observed in *FveSnRK2.1*‐OE and *FveWRKY50* transgenic strawberries (Figures 4F and 2C).

### 2.5 Anthracnose Triggers JA Signalling and Biosynthesis by Activating FveSnRK2.1/FveWRKY50 Regulatory Module

JA biosynthesis is typically regulated by a dynamic equilibrium system through feedback mechanisms (Cao et al. 2025). Positive feedback amplifies stress responses, while negative feedback prevents excessive signal activation to maintain homeostasis (Cao et al. 2025). To investigate whether anthracnose‐induced MeJA biosynthesis operates via positive or negative feedback, we treated diploid ‘di Bosco’ and octoploid ‘Benihoppe’ strawberries with exogenous MeJA. Phenotypic analyses revealed dose‐dependent increases in anthracnose susceptibility across three experimental models: detached leaves, attached leaves, and crown‐infected whole plants (Figure S8A–D). Moreover, exogenous MeJA treatment triggered significant accumulation of endogenous MeJA (Figure S8E). Consistently, exogenous application of 20 μM MeJA (under pathogen‐free conditions) transcriptionally activated *FveAOS2* and *FveAOC3*, which correlated with MeJA accumulation and enhanced disease susceptibility phenotypes (Figure S8E,F). These findings further validated the positive feedback regulation between JA signalling and MeJA biosynthesis during infection.

**FIGURE 5:**
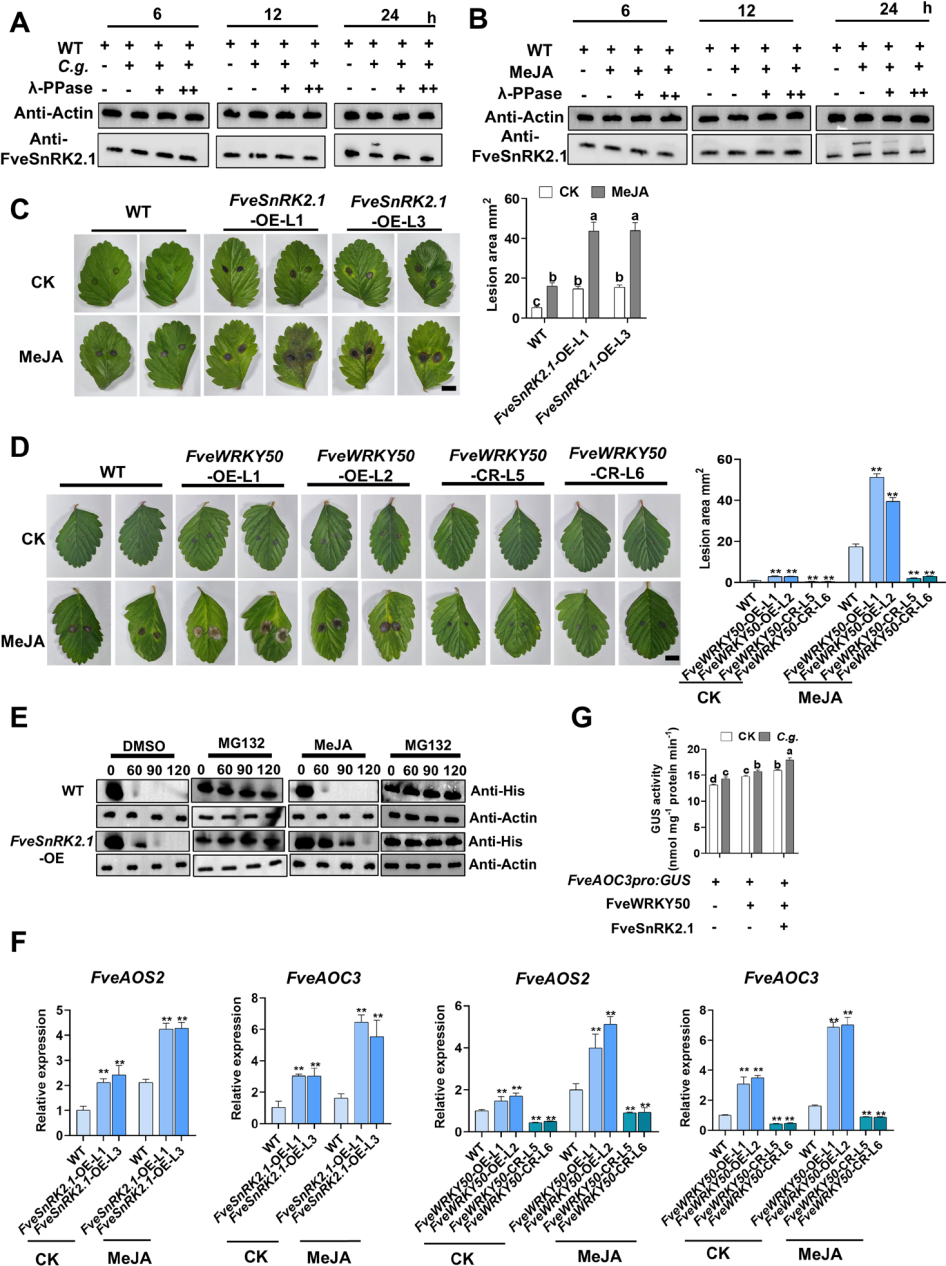
MeJA treatment enhances strawberry susceptibility to anthracnose by activating the FveSnRK2.1‐FveWRKY50‐*FveAOS2/FveAOC3* regulatory pathway. (A, B) Phos‐tag assay confirmed that FveSnRK2.1 phosphorylation was significantly activated by (A) *C. gloeosporioides* infection and (B) 100 μM MeJA treatment. Proteins were detected by western blot with anti‐FveSnRK2.1 antibody. λ‐PPase was used to verify the retarded mobility band was resulted by phosphorylation. (C, D) Phenotypic analysis and lesion area quantification in WT and *FveSnRK2.1*‐OE strawberry leaves (C) and WT, *FveWRKY50*‐ OE, and *FveWRKY50*‐CR strawberry leaves (D). Leaves were pre‐treated with 100 μM MeJA 24 h before anthracnose infection, and lesions were imaged at 5 dpi. Values are means ±SEM of three biological replicates (each with 10 technical replicates). (C) Different lowercase letters indicate significant differences (one‐way ANOVA, Tukey's test). (D) Statistical significance was determined by Student's *t* test (*p < 0.05, **p < 0.01). Bar, 1 cm. (E) Cell‐free degradation assay showing MeJA treatment enhanced His‐FveWRKY50 stability via FveSnRK2.1. Recombinant His‐FveWRKY50 proteins were incubated with total protein extracts from WT or *FvSnRK2.1*‐OE fruits in the presence of 50 μM MG132 or DMSO, followed by western blot with anti‐His antibody. (F) qRT‐PCR analysis of *FveAOS2* and *FveAOC3* expression in *C. gloeosporioides*‐infected WT and *FveSnRK2.1*‐OE leaves pre‐treated with MeJA (100 μM, 24 h prior to anthracnose infection) at 3 dpi. Values are means ±SEM of three biological replicates. Statistical significance was determined by Student's *t* test (*p < 0.05, **p < 0.01). (G) *C. gloeosporioides*‐induced *FveAOC3* expression was enhanced by FveSnRK2.1 and FveWRKY50. ‘Benihoppe’ fruits were co‐infiltrated with Agrobacterium carrying *FveSnRK2.1/FveWRKY50* effectors and *FveAOC3pro:GUS* reporter, then infected with anthracnose. GUS activity was assayed at 24 hpi (hours post‐anthracnose infection). Values are means ± s.d. (*n* = 3; 15 fruits/replicate). Different lowercase letters indicate significant differences (one‐way ANOVA, Tukey's test).

To elucidate functional connections within the FveSnRK2.1‐FveWRKY50 phosphorylation cascade during anthracnose infection, we demonstrated that both anthracnose infection and MeJA treatment increased FveSnRK2.1 phosphorylation using Phos‐tag assays (Figure 5A,B). Genetic evidence showed that, compared to WT, MeJA‐induced susceptibility was amplified in *FveSnRK2.1*‐OE and *FveWRKY50*‐OE lines, whereas CRISPR‐mediated knockout of *FveWRKY50* attenuated this effect (Figure 5C,D). Furthermore, MeJA treatment significantly enhanced FveSnRK2.1‐increased protein stabilisation of His‐FveWRKY50 (Figure 5E). These phenotypic variations and protein stability differences were consistent with the transcriptional activation of *FveAOS2* and *FveAOC3* in *FveSnRK2.1*‐OE and *FveWRKY50* transgenic strawberries upon MeJA treatment (Figure 5F). Using a strawberry fruit transient expression system, we further demonstrated that FveSnRK2.1 synergizes with anthracnose infection to enhance FveWRKY50‐mediated regulation of GUS activity driven by the *FveAOC3* promoter (Figure 5G). Collectively, these results establish a mechanistic link whereby anthracnose triggers JA signalling and biosynthesis through the FveSnRK2.1‐FveWRKY50‐*FveAOS2/FveAOC3* phosphorylation cascade.

### 2.6 Anthracnose and JA Signal Regulate the Stability of FveJAZ5 to Activate FveSnRK2.1/FveWRKY50‐Mediated Susceptibility in Strawberry

**FIGURE 6:**
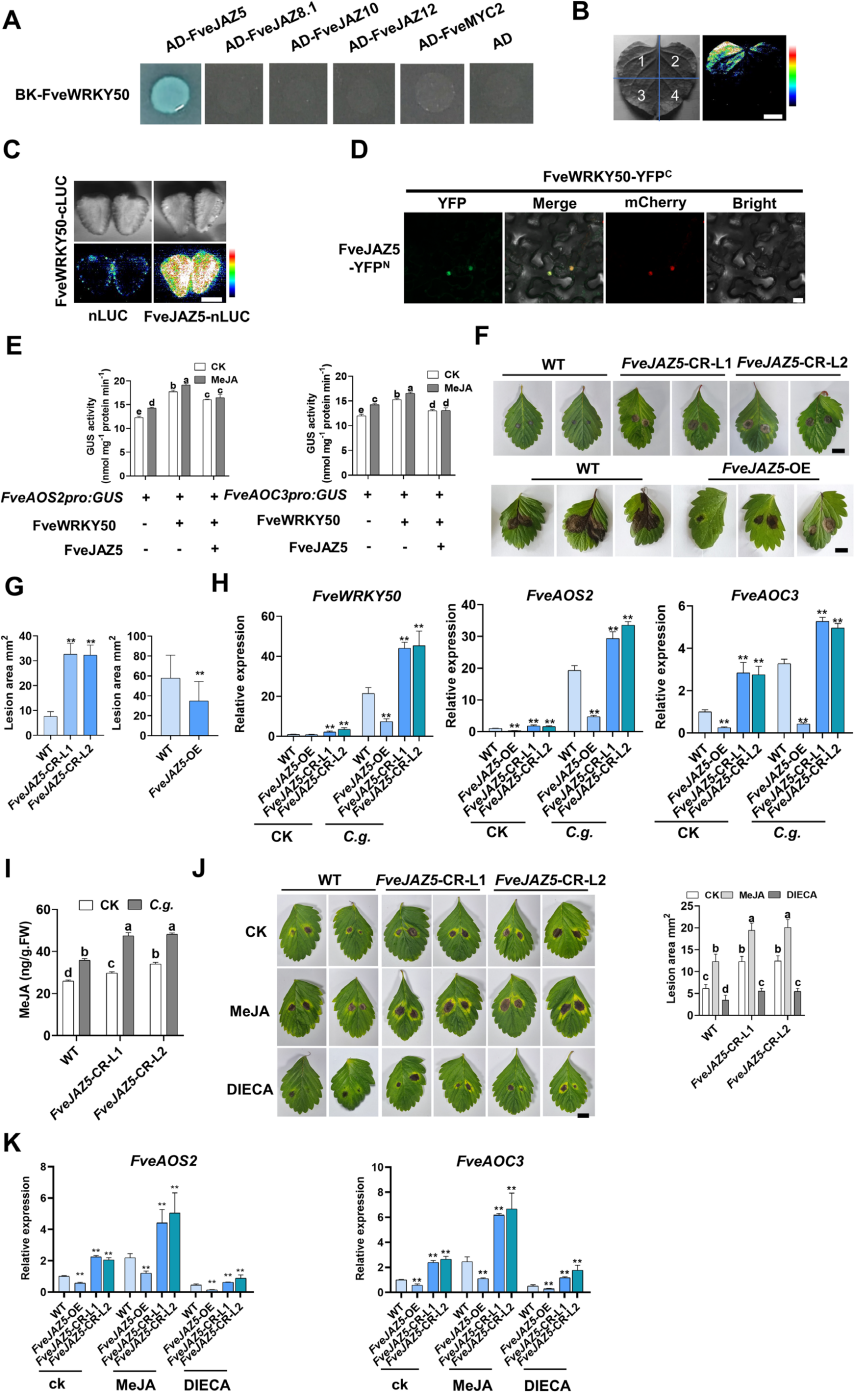
FveJAZ5 interacts with FveWRKY50 and modulates anthracnose resistance. (A) Y2H assay for interaction between FveWRKY50 and anthracnose‐responsive FveJAZ members (FveJAZ5/8.1/10/12) or FveMYC2. (B, C) LIC assay validated FveWRKY50‐FveJAZ5 interaction in strawberry fruits (B) and tobacco leaves (C). 1, FveWRKY50‐cLUC+nLUC‐FveJAZ5; 2, FveWRKY50‐cLUC+Nluc; 3, cLUC+nLUC‐FveJAZ5; 4, cLUC+nLUC. Scale bars, 1 cm. (D) BiFC assay detected FveWRKY50‐FveJAZ5 interaction in the nucleus of tobacco leaves. A nuclear marker pSuper::*NF‐YA4*‐mCherry with red fluorescence was used to visualise the cell nucleus; scale bars, 20 μm. (E) FveJAZ5 repressed MeJA‐triggered, FveWRKY50‐mediated upregulation of *FveAOS2* and *FveAOC3* expression. ‘Benihoppe’ fruits were co‐infiltrated with Agrobacterium carrying FveJAZ5/FveWRKY50 and *FveAOS2pro:GUS*/*FveAOC3pro:GUS*, treated with 100 μM MeJA, and GUS activity assayed at 24 hpi. Values are means ± s.d. (*n* = 3; 15 fruits/replicate). (F, G) Phenotypic analysis (F) and lesion area quantification (G) in *C. gloeosporioides*‐infected WT, *FveJAZ5*‐ OE, and *FveJAZ5*‐CR strawberry leaves 5 dpi. Values are means ±SEM of three biological replicates (each with 10 technical replicates). Statistical significance was determined by Student's *t* test (*p < 0.05, **p < 0.01). Bar, 1 cm. (H) Expression of *FveWRKY50*, *FveAOS2* and *FveAOC3* in *C. gloeosporioides*‐infected WT, *FveJAZ5*‐ OE and *FveJAZ5*‐CR leaves at 3 dpi, as measured by qRT‐PCR. Values are means ±SEM of three biological replicates. Statistical significance was determined by Student's *t* test (*p < 0.05, **p < 0.01). (I) MeJA content in WT and *FveJAZ5*‐CR strawberry leaves infected with *C. gloeosporioides* at 3 dpi. Values are means ±SEM of three biological replicates. Different lowercase letters indicate significant differences (one‐way ANOVA, Tukey's test). (J) Detection of phenotype and lesion area in WT and *FveJAZ5*‐CR strawberry leaves. Leaves were pre‐treated with 100 μM MeJA or 50 μM DIECA 24 h before anthracnose infection, and lesions were imaged at 5 dpi. Values are means ±SEM of three biological replicates (each with 10 technical replicates). Different lowercase letters indicate significant differences (one‐way ANOVA, Tukey's test). Bar, 1 cm. (K) qRT‐PCR analysis of *FveAOS2* and *FveAOC3* expression in *C. gloeosporioides*‐infected WT, *FveJAZ5*‐ OE and *FveJAZ5*‐CR leaves at 3 dpi, treated as in (I). Values are means ± s.d. (*n* = 3 biological replicates, each with 3 technical replicates).

In the JA signalling network, MYC2 and JAZ proteins play central roles in feedback regulation (Garrido‐Bigotes et al. 2018; Cao et al. 2025; Roychowdhury et al. 2025; Verma et al. 2025). RNA‐Seq and RT‐qPCR analyses revealed that anthracnose infection significantly induced the expression of *FveJAZ5*, *FveJAZ8.1*, *FveJAZ10*, and *FveJAZ12* (Figure S9A,B; Data S4). Previous studies have established that while JAZ proteins lack intrinsic transcriptional activity, they often function as co‐transcriptional regulators to modulate downstream gene expression. Therefore, we examined the interaction between FveWRKY50 and FveJAZ proteins. Although *FveMYC2* expression remained unaffected by anthracnose infection, we further explored its interaction with FveWRKY50 due to MYC2's pivotal regulatory role in the JA signalling pathway. The results demonstrated that only FveJAZ5 interacted with FveWRKY50 in yeast cells (Figure 6A). Subsequent analyses confirmed this interaction in the nuclei of tobacco leaves and strawberry fruits (Figure 6B–D). Further analysis showed that FveJAZ5 repressed FveWRKY50‐mediated activation of GUS driven by the *FveAOS2* and *FveAOC3* promoters; however, this repressive effect was attenuated by MeJA treatment (Figure 6E), indicating that MeJA diminishes FveJAZ5's inhibitory activity. Collectively, these results position FveJAZ5 as a transcriptional repressor of FveWRKY50‐activated JA biosynthetic genes, establishing it as a key negative regulator of anthracnose‐induced MeJA accumulation and disease susceptibility.

To validate the proposed regulatory mechanism, we generated *FveJAZ5*‐overexpressing (*FveJAZ5*‐OE) and CRISPR knockout (*FveJAZ5*‐CR) strawberry lines (Figure S9C,D). Phenotypic analysis revealed that *FveJAZ5‐CR* mutants exhibited increased susceptibility to anthracnose, whereas *FveJAZ5‐OE* plants displayed reduced susceptibility compared to wild‐type strawberries (Figure 6F,G). Consistently, anthracnose‐induced expression of *FveWRKY50*, *FveAOS2*, and *FveAOC3* was significantly attenuated in *FveJAZ5‐OE* plants but strikingly upregulated in *FveJAZ5‐CR* mutants (Figure 6H). Further pharmacological experiments demonstrated that MeJA and DIECA showed more pronounced effects on both anthracnose susceptibility and endogenous MeJA accumulation in *FveJAZ5‐CR* mutants relative to WT (Figure 6I,J). Consistent with this, MeJA‐induced expression of *FveAOS2* and *FveAOC3* was significantly reduced in *FveJAZ5‐OE* lines but markedly enhanced in *FveJAZ5‐CR* mutants (Figure 6K). Conversely, the inhibitory effect of DIECA on *FveAOS2* and *FveAOC3* expression was potentiated in *FveJAZ5‐OE* plants but diminished in *FveJAZ5‐CR* mutants (Figure 6K). These results align with our earlier GUS activity assay, where FveJAZ5 repressed *FveWRKY50*‐mediated activation of *FveAOS2* and *FveAOC3* promoters (Figure 6E). Taken together, these data establish that *FveJAZ5* acts as an important negative regulator of JA‐mediated susceptibility to anthracnose in strawberry: by interacting with FveWRKY50, *FveJAZ5* suppresses FveWRKY50's transcriptional activation of *FveAOS2* and *FveAOC3*, thereby modulating JA accumulation and anthracnose susceptibility.

**FIGURE 7:**
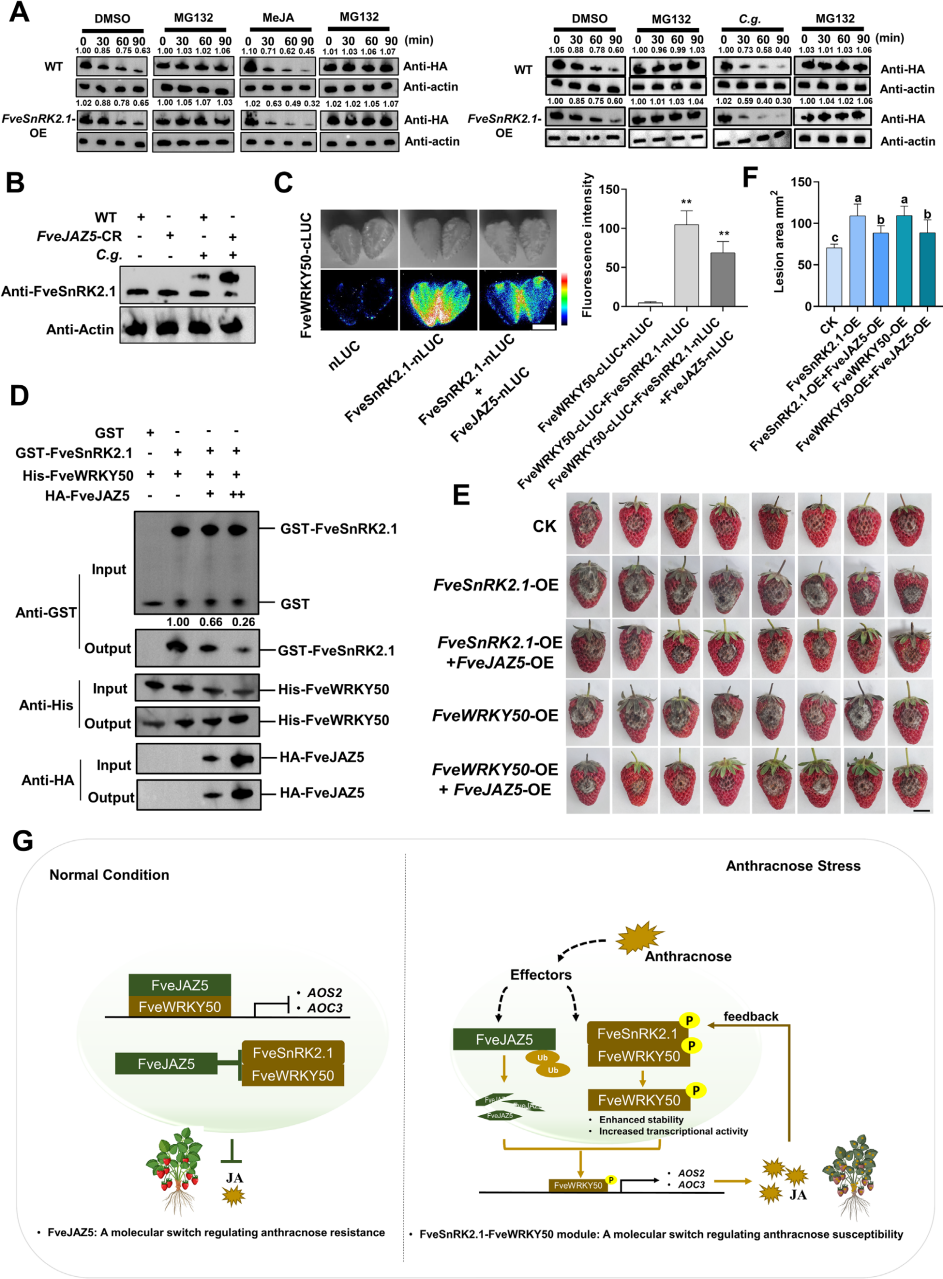
FveJAZ5 regulates anthracnose resistance in strawberry by fine‐tuning the FveSnRK2.1–FveWRKY50 module. (A) Cell‐free degradation analysis showing MeJA/anthracnose treatment decreased HA‐FveJAZ5 protein stability via FveSnRK2.1. Recombinant HA‐FveJAZ5 was incubated with fruit protein extracts (WT or FveSnRK2.1‐OE) in 50 μM MG132 or DMSO at 37°C for 90 min, detected by anti‐HA antibody. (B) Phos‐tag assay confirmed that FveSnRK2.1 phosphorylation activated by *C. gloeosporioides* was enhanced in *FveJAZ5*‐CR fruits. Proteins were detected by western blot with anti‐FveSnRK2.1 antibody. (C) LCI assay validated FveJAZ5 repressed FveWRKY50‐FveSnRK2.1 interaction in strawberry fruits. Luciferase activity was quantified by ImageJ; values are means ± s.d. (*n* = 3; 15 fruits/replicate). *p < 0.05, *p < 0.01 (Student's *t*‐test). Bar, 1 cm. (D) Pull‐down analysis confirming HA‐FveJAZ5 repressed the interaction between GST‐FveWRKY50 and His‐FveSnRK2.1. Purified His‐FveWRKY50 protein was incubated with GST or GST‐FveSnRK2.1, adding different amounts of HA‐FveJAZ5. Ni‐NTA agarose was used for immunoprecipitation. Recombinant proteins GST‐FveSnRK2.1, His‐FveWRKY50, and HA‐FveJAZ5 were detected by western blot using anti‐GST, anti‐His and anti‐HA antibodies; GST protein alone served as a negative control. (E, F) Overexpression of *FveJAZ5* reduced FveSnRK2.1/FveWRKY50‐mediated anthracnose susceptibility in ‘Benihoppe’ fruits. Lesions were imaged and quantified at 5 dpi (F). Values are means ±SEM of three biological replicates. Different lowercase letters indicate significant differences (one‐way ANOVA, Tukey's test). Bar, 1 cm. (G) A proposed model for JA‐mediated susceptibility to anthracnose in strawberry. Physiological state: Endogenous JA maintains basal levels to regulate plant growth. FveJAZ5 interacts with FveWRKY50 to form a repressor complex, inhibiting expression of JA biosynthesis genes *FveAOS2* and *FveAOC3*. Meanwhile, FveJAZ5 blocks FveWRKY50‐FveSnRK2.1 interaction, preventing regulatory complex formation. Anthracnose stress: Activated FveSnRK2.1 triggers dual events: I, Ubiquitination‐mediated degradation of FveJAZ5 releases FveWRKY50; II, Phosphorylation of FveWRKY50 enhances its stability and activity. This upregulates *FveAOS2/FveAOC3*, promoting JA biosynthesis. JA then amplifies the FveSnRK2.1‐FveWRKY50 phosphorylation module via a positive feedback loop, sustaining JA production and increasing disease susceptibility. Solid arrows denote the research findings established in this study, whereas dashed arrows indicate the aspects yet to be elucidated in the future studies.

As MeJA was previously shown to diminish the repressive effects of FveJAZ5 on the FveWRKY50‐*FveAOS2/FveAOC3* module (Figure 6E), we next investigated whether and how FveJAZ5 interfaces with the FveSnRK2.1‐FveWRKY50 cascade during JA‐mediated susceptibility to anthracnose in strawberry. To this end, we examined the impact of MeJA and anthracnose infection on FveSnRK2.1‐mediated degradation of FveWRKY50. Results revealed that both anthracnose infection and MeJA treatment promoted HA‐tagged FveJAZ5 degradation in a 26S proteasome‐dependent manner (Figure 7A). Further analysis of *FveJAZ5*‐CR mutants demonstrated that anthracnose‐triggered phosphorylation of FveSnRK2.1 was significantly enhanced compared to wild‐type controls (Figure 7B), indicating that FveJAZ5 acts as a negative regulator of anthracnose‐induced FveSnRK2.1 phosphorylation. Although FveSnRK2.1 did not directly interact with FveJAZ5, co‐expression assays revealed that FveJAZ5 attenuated the FveSnRK2.1‐FveWRKY50 interaction in both strawberry fruit cells and in vitro systems (Figure 7C,D). Consistent with this, transient overexpression of *FveJAZ5* in strawberry fruits suppressed FveSnRK2.1/FveWRKY50‐mediated susceptibility (Figure 7E,F; Figure S9E), further confirming FveJAZ5's repressive role in modulating the FveSnRK2.1‐FveWRKY50 module during anthracnose infection.

Taken together, under normal conditions, FveJAZ5 interacts with FveWRKY50 to repress transcriptional activation of *FveAOS2* and *FveAOC3*, avoiding the excessive activation of MeJA biosynthesis. Upon anthracnose infection, FveJAZ5 undergoes proteasomal degradation, releasing FveWRKY50. Concurrently, anthracnose activates FveSnRK2.1, which phosphorylates FveWRKY50 to enhance transcription of *FveAOS2* and *FveAOC3*, leading to elevated MeJA accumulation. The induced MeJA then acts as a signalling molecule to activate the FveSnRK2.1–FveWRKY50–*FveAOS2*/*FveAOC3* pathway, amplifying anthracnose susceptibility. MeJA‐triggered FveJAZ5 degradation further reinforces this positive feedback loop, modulating strawberry disease susceptibility (Figure 7G). Both CRISPR‐edited *FveWRKY50* knockout and *FveJAZ5* overexpression generate strawberry germplasm with enhanced resistance (Figures 2 and 6).

## 3 Discussion

The identification of resistance (R) genes and susceptibility (S) genes is of great significance for breeding disease‐resistant varieties. In previous breeding practices, the introduction of *R* genes has long served as a primary focus. It should be noted that the disease resistance efficacy of *R* genes can be influenced by the evolutionary dynamics of pathogen effectors and may entail adaptive trade‐offs in certain contexts – such as growth–defence trade‐offs. Concurrently, the strategy targeting *S* genes has emerged in recent years as an innovative approach, demonstrating substantial potential for achieving durable resistance (Li et al. 2020; Koseoglou et al. 2022; Han, Li, et al. 2025; Ikram et al. 2025; Wang et al. 2025). For instance, the knockout of *MLO* genes in wheat has conferred broad‐spectrum resistance to powdery mildew without compromising yield (Li, Lin, et al. 2022), and similar success has been achieved in wheat by targeting *TaPsIPK1*, which enhances stripe rust resistance without affecting yield (Wang et al. 2022). Especially, the emergence of genome‐editing technologies, particularly CRISPR‐Cas9, has revolutionised crop improvement by enabling precise modifications of disease‐related genes (Li et al. 2020; Li, Lin, et al. 2022; Koseoglou et al. 2022; Wang et al. 2022, 2025; Han, Li, et al. 2025). While this emerging strategy offers unique advantages, *R* genes retain significant value in disease resistance improvement. Both approaches hold distinct value for crop disease resistance breeding across diverse scenarios. Nevertheless, both *R* genes and *S* genes require further identification, and molecular mechanisms underlying anthracnose pathogenesis remain largely uncharacterized, thereby impeding the application of genome‐editing strategies for resistance breeding in anthracnose‐infected crops.

In this study, we identified *FveWRKY50* and *FveSnRK2.1* as *S* genes for anthracnose infection in strawberry, while *FveJAZ5* functioned as an *R* gene (Figures 1, 4 and 6). Through stable genetic transformation in the diploid cultivar ‘di Bosco’ and transient expression assays in the octoploid cultivar ‘Benihoppe’, we demonstrated that their regulatory roles in anthracnose response are conserved across diverse tissues (root, leaf, fruit, crown) and ploidy levels (Figures 1 and 7E). This conservation is further supported by previous findings demonstrating that *FaWRKY1*, the homologous gene of *FveWRKY50* in octoploid strawberry, functions as a negative regulator of *C. acutatum* ‐induced anthracnose in octoploid ‘Primoris’, as revealed by transient expression analysis in fruits (Encinas‐Villarejo et al. 2009; Higuera et al. 2019). Notably, the functional equivalence of *FveWRKY50* and *FaWRKY1* across diploid and octoploid genetic backgrounds suggests a conserved susceptibility module in strawberry–anthracnose interactions. We further identified *FveMYB108* as an upstream transcriptional regulator of *FveWRKY50* expression (Figure S2). Methodologies such as yeast one‐hybrid (Y1H) have proven effective in uncovering upstream transcriptional regulators; applying these approaches to the promoters of *FveWRKY50* or *FveMYB108* may facilitate the identification of additional key transcriptional components and help complete the regulatory networks in future studies. Together, these results demonstrate that despite genetic differences in anthracnose resistance among strawberry cultivars, key regulatory genes may still maintain conserved signalling pathways across different tissue types, cultivars and ploidy levels. This indicates the existence of a relatively stable regulatory mechanism that plays an essential role in ensuring effective resistance responses under diverse genetic backgrounds and tissue contexts. Such conservation provides a mechanistic basis for leveraging diploid strawberries in functional genomics studies, thus facilitating the efficient identification of translatable gene resources for breeding programs.

Our study further revealed that FveJAZ5, FveWRKY50 and FveSnRK2.1 participate in strawberry anthracnose regulation through a hierarchical network involving transcriptional regulation, ubiquitination modification, and phosphorylation modification (Figure 7G). Given the significant variation in anthracnose resistance among strawberry cultivar groups (e.g., European‐American vs. Asian cultivars), it is intriguing to investigate whether the coding genes of these proteins represent potential targets of domestication selection. Specifically, do genomic structural variations or single nucleotide polymorphisms (SNPs) in these genes lead to differences in transcriptional and post‐transcriptional regulatory mechanisms, thereby contributing to cultivar‐specific resistance disparities? Previous studies have identified *a TIFY 11A‐like protein*, the homologous gene of *FveJAZ5* in octoploid strawberry, as a candidate gene within the *FaRCg1* genomic region, which confers resistance to C. *gloeosporioides* in different cultivars (Salinas et al. 2019; Chandra et al. 2021). *FveAOS2* and *FveAOC3* were identified as key JA biosynthesis genes induced by anthracnose (Figure S4; Data S4); however, whether their expression levels vary among different cultivars and how these variations correlate with differences in resistance remain to be fully elucidated. From the perspective of crop domestication and improvement, dissecting the domestication basis of this regulatory module and its improvement potential using the rich diversity of anthracnose resistance in strawberry varieties will provide critical theoretical insights for breeding programs.

The trade‐off between disease resistance and fitness remains a central challenge in plant breeding, highlighting the importance of isolating resistance genes that are not associated with fitness penalties for sustainable crop improvement (Li et al. 2020; Koseoglou et al. 2022; Han, Salinas, et al. 2025; Wang et al. 2025). Achieving this objective necessitates the precise identification of pathway‐specific regulatory factors and the development of isogenic knockout materials. In this study, we show that targeted disruption of *FveWRKY50* and overexpression of *FveJAZ5* both enhance anthracnose resistance in strawberry (Figures 1 and 6). Our previous research demonstrated that heterozygous (*−114/−5*) and homozygous (*−114/−114*) *FveWRKY50* mutants exhibit pleiotropic phenotypes, including early flowering and delayed fruit ripening, with the *−114/−114* homozygotes displaying severe developmental defects (Chen et al. 2023). Notably, in the T2 generation of homozygous *FveWRKY50* mutants, the newly identified line (*−5/−5* homozygotes) and −5/−114 heterozygotes used in this study exhibited enhanced anthracnose resistance and early flowering without any observable developmental abnormalities (Figure S1F). These findings suggest that locus‐specific editing of *FveWRKY50* may facilitate the development of elite strawberry varieties that combine improved disease resistance with favourable agronomic traits. Interestingly, *FveJAZ5*‐OE plants also displayed early flowering and increased resistance (Figure S9F). Whether these two traits are genetically linked or if JA signalling independently influences flowering time remains to be determined. Taken together, these results establish a molecular framework for precision breeding strategies aimed at decoupling disease resistance from developmental trade‐offs in strawberry.

Phosphorylation is a well‐established regulatory mechanism in plant disease resistance signalling and has been widely reported across multiple crop species (Jiang et al. 2022). For instance, the effector protein CfEC92 secreted by *C. fructicola* in apple activates the MdNDPK‐MdMAPK3 phosphorylation cascade, which mediates resistance via regulation of the SA pathway (Liu et al. 2025). Studies in *Arabidopsis* and *tobacco* also highlight the important role of the MAPK family in anthracnose responses (Zhou et al. 2023). However, the involvement of the plant‐specific SnRK2 kinase family in regulating anthracnose resistance remains largely unexplored. The functions of SnRK2 kinases are closely linked to their subfamily classification (Mondal et al. 2024). Most previous research has focused on subfamily III members, particularly their roles in abscisic acid (ABA) signalling and abiotic stress responses (Mondal et al. 2024). In contrast, FveSnRK2.1, the focus of this study, belongs to subfamily II, which remains less characterised in terms of function. Using yeast two‐hybrid and phosphorylation assays, we confirmed that FveSnRK2.1 specifically phosphorylates FveWRKY50 and modulates its activity (Figures 3 and 5G). Further functional analysis revealed that phosphorylation at Serine‐88 is important, though not exclusively responsible, for FveWRKY50‐mediated susceptibility (Figure 3F,G), suggesting that additional phosphorylation sites may contribute to its regulation. Notably, AtSnRK2.8, a subfamily II member in *Arabidopsis*, participates in signalling by phosphorylating the SA receptor AtNPR1 (Lee et al. 2015). Our results further showed that JA and SA levels change in parallel in *FveWRKY50*‐OE lines (Figure S3A). Given that *FveWRKY50* overexpression significantly increases strawberry susceptibility, which is consistent with the promoting effect of JA, we focus on how the FveSnRK2.1‐FveWRKY50 module regulates the JA signalling pathway (Figure 5). Although our analysis centers on the JA pathway, the crosstalk among ABA, SA and JA signalling in strawberry anthracnose resistance remains to be fully understood. Of particular interest is the potential interaction between JA and ABA signalling, which is a key hormone regulating fruit ripening and quality, and this may offer a new strategy for simultaneously improving both disease resistance and fruit quality (Li, Grierson, et al. 2022).

Another unresolved aspect is how environmental temperature influences anthracnose resistance. Our previous work demonstrated that low temperature suppresses anthocyanin accumulation in strawberry fruits through activation of the FveSnRK2.6‐FveMAPK3 and FveMAPK3‐FveWRKY50 phosphorylation cascades (Mao et al. 2022; Chen et al. 2023). In this study, under the temperature conditions tested, *FveMAPK3* overexpression did not significantly alter anthracnose susceptibility (Figure S2). However, whether SnRK2‐MAPK modules contribute to temperature‐modulated immunity remains an important avenue for future investigation. Finally, the differential kinase interactions observed for WRKY50, phosphorylated by SnRK2.1 in strawberry but typically by MAPKs in other species, raise the question of whether this reflects pathogen‐specific or species‐specific regulatory adaptations. Clarifying this distinction will be essential for understanding the evolutionary context of WRKY50 regulation.

Effector proteins serve a critical role in plant‐pathogen interactions, yet the identification of effector proteins from strawberry anthracnose pathogens remains significantly understudied. Studies in apple have demonstrated that *C. fructicola* effectors CfEC12, CfEC28 and CfEC92 exhibit diverse functions: CfEC12 regulates SA‐mediated resistance by interacting with the NPR1 regulatory factor MdNIMIN (Shang, Liu, et al. 2024); CfEC28 modulates resistance by targeting DAHPS, the rate‐limiting enzyme of the shikimate pathway, thereby influencing secondary metabolism (Shang, Liang, et al. 2024); and CfEC92 mediates SA‐dependent resistance through interaction with the protein kinase MdNDPK (Liu et al. 2025). These findings highlight the functional diversity of effectors and the complexity of host susceptibility regulation. Moreover, previous studies suggest that JAZ proteins may serve as effector interaction targets (Jiang et al. 2013; Gimenez‐Ibanez et al. 2014), underscoring the importance of identifying strawberry anthracnose effectors and investigating their potential interactions with key regulators such as FveSnRK2.1 and FveJAZ5. Notably, JA exhibits contrasting regulatory roles in anthracnose responses across plant species: it positively regulates resistance in apple and mango but negatively in strawberry, maize, and tea (Amil‐Ruiz et al. 2016; Gorman et al. 2020; Fang et al. 2021; Huang et al. 2023; Jeyaraj et al. 2023; Ma et al. 2023; Lv et al. 2024). In this study, we demonstrate that JA acts as a central positive regulator of anthracnose susceptibility in strawberry (Figure S8). This is consistent with previous observations showing a negative correlation between JA levels and anthracnose resistance in functional studies of *FaHSP17.4* and *FaMBL1* (Fang et al. 2021; Ma et al. 2023). Is this interspecific variation linked to effector‐mediated signal recognition? How do plants perceive effectors across different species? Addressing these questions is essential for understanding the mechanisms underlying broad‐spectrum disease resistance. Furthermore, given JA's well‐established positive role in necrotrophic diseases (e.g., grey mould, insect pests) (Cao et al. 2025; Roychowdhury et al. 2025; Verma et al. 2025), comparative analyses of JA function in necrotrophic versus hemibiotrophic pathogens, along with the identification of specific responsive genes, will provide precise molecular targets for coordinated resistance improvement.

As a core component of JA signalling, this study confirms that *FveJAZ5* acts as a key ‘brake’ gene suppressing strawberry anthracnose susceptibility (Figure 7G). Mechanistically, FveJAZ5 fine‐tunes the kinase activity of FveSnRK2.1 and modulates the FveSnRK2.1‐FveWRKY50 regulatory module to control the “on–off” switch of disease susceptibility (Figure 7B–F). This uncovers a previously unrecognised regulatory layer within the JA signalling cascade, wherein a JAZ protein interfaces with an SnRK2 kinase to gatekeep a WRKY‐dependent susceptibility program. Moreover, ubiquitin‐mediated degradation of FveJAZ5, induced by both the pathogen and MeJA, constitutes the central regulatory mechanism (Figure 7A). Notably, although FveJAZ5 transcription is pathogen‐induced, its protein level is degraded, with phenotypic outcomes aligning precisely with protein changes (Figures 6F and 7A; Figure S9B). The discrepancy between transcriptional induction and post‐translational degradation suggests a more complex regulatory mechanism. Do differences in basal expression levels, synthesis rates or degradation kinetics correlate with cultivar resistance? Which factors are most critical? Could these traits be developed into molecular markers? Do variations in SnRK2.1 phosphorylation levels exist among cultivars, and how do they relate to FveJAZ5 dynamics? These scientific questions warrant further investigation.

In canonical JA signalling, JAZ degradation depends on COI1‐mediated ubiquitination (Cao et al. 2025; Roychowdhury et al. 2025; Verma et al. 2025). However, whether COI1 participates in FveJAZ5 degradation triggered by the anthracnose pathogen remains to be determined. Furthermore, FveMYC2, a central transcriptional activator in classical JA signalling, was neither transcriptionally induced by the pathogen and nor did it interact with FveWRKY50 at the protein level (Figure 6A; Figure S9B), suggesting that the JA signalling pathway activated during anthracnose infection may diverge from the canonical COI1–JAZ–MYC2 module. These findings highlight the importance of further elucidating the similarities and differences between the FveJAZ5‐mediated susceptibility pathway identified in this study and the conserved JA signalling pathways during anthracnose infection. Moreover, the incomplete restoration of resistance by MeJA in the *FveWRKY50*‐CR mutant (Figure 5D), combined with the direct interaction between FveWRKY50 and FveJAZ5 (Figure 6A–D), suggests that FveWRKY50 functions directly in JA signal transduction rather than merely regulating JA biosynthesis. Furthermore, the partial recovery of resistance suggests that FveWRKY50 may also modulate susceptibility through JA‐independent pathways, although this mechanism requires further experimental validation. Meanwhile, other members of the strawberry WRKY family, besides FveWRKY50, were also significantly upregulated by anthracnose. Although single‐gene editing confirmed the central roles of *FveJAZ5* and *FveWRKY50*, the incomplete suppression of JA biosynthesis in *FveWRKY50* knockout mutants implies potential cooperative or redundant regulatory mechanisms within the WRKY family (Figure 2A). Investigating whether modulating the collective responsiveness of WRKY members can yield strawberry lines with enhanced or even complete resistance represents a promising direction for future research.

Given the substantial variation in anthracnose resistance among strawberry cultivars, future studies could integrate the regulatory module identified here with evolutionary and breeding genetics approaches to dissect its evolutionary basis and breeding potential. This would establish a theoretical foundation for developing new strawberry varieties with coordinated improvements in broad‐spectrum resistance and agronomic performance.

## 4 Materials and Methods

### 4.1 Plant Materials and Growth Conditions

Octoploid strawberry *Fragaria × ananassa* Duch. cv ‘Benihoppe’ and diploid strawberry *F. vesca* cv ‘Fragola di Bosco’ were used in this study. Strawberries were grown under a 12 h light/12 h dark photoperiod, with a light intensity of 200–300 μmol m^−2^ s^−1^, day/night temperatures of 25°C/15°C, and a relative humidity of 70%.

### 4.2 Artificial Inoculations With *C. fructicola*

*C. fructicola* (*Colletotrichum gloeosporioides species complex*) has recently been identified as a more aggressive anthracnose pathogen in strawberry production systems. The *C. fructicola* strain used in this study was kindly provided by Prof. Qinghua Gao (Zhang et al. 2018). For inoculation, the spore suspension concentration was adjusted to 1 × 10^7^ cfu/mL for strawberry leaves and crowns, and 3 × 10^6^ cfu/mL for fruits. All inoculated materials were subsequently incubated at 28°C under conditions of 100% relative humidity and a 12 h light/12 h dark cycle. All experiments were repeated three times, with at least 10 samples per material in each replicate. The lesion area was measured using ImageJ software. Petiole death was determined when blackening and wilting occurred at the base followed by detachment from the plant. The petiole mortality rate was calculated as the number of necrotic petioles divided by the total number of petioles.

### 4.3 Quantification of Phytohormones

Hormone quantification was performed using enzyme‐linked immunosorbent assay (ELISA) and liquid chromatography–tandem mass spectrometry (LC–MS/MS). The collected samples were immediately frozen in liquid nitrogen and subsequently analysed by Pronets testing (Wuhan, China).

### 4.4 RNA Sequencing

Total RNA was extracted from water‐treated and *C. fructicola* ‐infected strawberry seedlings using the E.Z.N.A. Total RNA Kit (Omega, Bienne, Switzerland). The RNA samples were then submitted to Novogene (Beijing, China) for sequencing. Sequencing was performed on an Illumina Novaseq platform with 150‐bp paired‐end reads. Reads were mapped to the *F. vesca* subsp. vesca reference genome (https://www.rosaceae.org/rosaceae_downloads/Fragaria_vesca/Fvesca‐genome.v4.0.a1/assembly/Fragaria_vesca_v4.0.a1.fasta.gz). Differentially expressed genes were identified based on DESeq2 analysis, with criteria of |log2 fold change| > 1 and adjusted *p*‐value (*p*_adj_) < 0.05.

### 4.5 RT‐qPCR Analysis

Total RNA from strawberries was extracted using the E.Z.N.A. Total RNA Kit (Omega, Bienne, Switzerland). cDNAs were obtained using M‐MLV reverse transcriptase (Promega, Madison, WI, USA). qPCR was performed on a real‐time PCR instrument (QuantStudio 6 Flex, Applied Biosystems, Thermo Fisher Scientific, USA) using Taq Pro Universal SYBR qPCR Master Mix (q712 Vazyme, Nanjing, China). *FveACTIN* was used as the internal reference of strawberry, and the relative gene expression was calculated by the $2^{-\Delta \Delta C_{T}}$ method. The primer sequences are listed in Data S6.

### 4.6 Transient Gene Expression in Strawberry Fruit

The coding sequences of *FveAOC3*, *FveWRKY50, FveWRKY50* ^*S88A*^ (FveWRKY50 phospho‐deficient mutant), *FveJAZ5, FveSnRK2.1* and *FveMYB108*, cloned from strawberry cDNA, were inserted into the pH7WG2D vector using the Gateway method and subsequently transformed into Agrobacterium EHA105 (Zhang et al. 2014). The pH7WG2D vector carried eGFP as a detection marker to evaluate transient transformation efficiency. Activated Agrobacterium cultures were collected, re‐suspended in infiltration buffer and incubated at 28°C for 2 h. The suspension was then injected into large green fruits of octoploid ‘Benihoppe’ strawberries using a 1‐mL syringe. After 3 days of incubation at 25°C, the fruits were harvested and either frozen in liquid nitrogen or inoculated with *C. fructicola* . The expression levels of target genes were quantified by RT‐qPCR. Primer sequences are provided in Data S6 and S7.

### 4.7 GUS Activity Assay

The GUS activity assay was conducted as previously described (Mao et al. 2022). The full‐length coding sequences of *FveWRKY50*, *FveWRKY50* ^*S88A*^, *FveJAZ5* and *FveSnRK2.1* were cloned into the pCAMBIA1301 vector under the control of the 35S promoter. The promoter regions of *FveAOC3* and *FveAOS2* were amplified and inserted upstream of the *GUS* reporter gene in the pCAMBIA1301 vector. The constructed vectors were transformed into *Agrobacterium tumefaciens* strain EHA105 and cultured at 28°C. Agrobacterium cultures were resuspended in infiltration buffer (10 mM MES, 10 mM MgCl_2_, 200 μM acetosyringone, pH 5.6 adjusted with NaOH) to an OD₆₀₀ of 0.6. Large green ‘Benihoppe’ fruits were injected using a 1‐mL needleless syringe. Infiltrated fruits were incubated at 25°C for 3 days. GUS activity was measured according to the protocol of Wei et al. (2018). Three independent biological replicates were performed, each using 10 fruits from different plants. Primers used for vector construction are listed in Data S7.

### 4.8 Stable Transformation of Diploid Strawberry

The CDS sequences of *FveWRKY50*, *FveSnRK2.1*, *FveJAZ5* and *FveMAPK3* were cloned from strawberry cDNA and subsequently inserted into the pH7WG2D vector using the Gateway method. The CRISPR vector was constructed following previously described methods (Zeng et al. 2018). Two specific sgRNAs targeting the CDS regions of *FveWRKY50* and *FveJAZ5* were designed and ligated into the pYLCRISPR/Cas9 vector. The constructed overexpression and gene‐editing vectors were then transferred into Agrobacterium EHA105 and used for stable transformation of diploid strawberries via the leaf disk method (Oosumi et al. 2006). Transformed plants were selected and cultured on medium supplemented with 2 mg/L hygromycin. Overexpression of the target genes was confirmed by detecting green fluorescence from eGFP and by RT‐qPCR analysis of relative gene expression levels. No GFP signal was detected in wild‐type (WT) samples. To identify edited plants, genomic DNA was extracted from transformed lines, and PCR‐amplified fragments encompassing the target sites were sequenced. Primer sequences and sgRNA designs are provided in Data S7.

### 4.9 Pharmacological Experiment

Strawberry plants were subjected to foliar spraying with MeJA (Sigma‐Aldrich, St. Louis, MO 63103, USA; Catalogue No. 392707) and DIECA (Rio British Time Technology Co. Ltd., Beijing, China) until runoff occurred. In contrast, control plants were sprayed with ddH_2_O only.

### 4.10 Two‐Yeast Hybrid Assay (Y2H)

GAL4‐based Two‐Hybrid System 3 (Clontech, Mountain View, CA, USA) was used for yeast double‐hybrid. The CDS of *FveWRKY50*, *FveSnRK2.1*, *FveSnRK2.2*, *FveSnRK2.3*, *FveSnRK2.4*, *FveSnRK2.5*, *FveSnRK2.6*, *FveSnRK2.7*, *FveSnRK2.8*, *FveSnRK2.9*, *FveJAZ5*, *FveJAZ8.1*, *FveJAZ10*, *FveJAZ12* and *FveMYC2* was cloned and connected to pGBKT7 or pGADT7 carriers, respectively. The combined plasmid was co‐transformed into AH109 strain and cultured on the medium of ‐Leu‐Trp and ‐Leu‐Trp‐His‐Ade. After 3 days, the plaque was stained with X‐α‐gal. Primers are listed in Data S7.

### 4.11 Luciferase Complementary Imaging Assay (LCI)

The CDS sequences of *FveWRKY50*, *FveSnRK2.1* and *FveJAZ5* were constructed on pCAMBIA1300‐cLUC vector and pCAMBIA1300‐nLUC vector, respectively. The constructed vectors were transferred into Agrobacterium EHA105 and cultured at 28°C. Resuspended Agrobacterium with infiltrating buffer (10‐mM MES pH 5.6, 10‐mM MgCl_2_ and 200‐μM acetosyringone) to OD600 of 0.6. After incubation at 28°C for 2 h, the suspension was mixed 1:1 and then injected into octoploid strawberry big green fruit or tobacco leaves. Fluorescence was examined after 3 days of incubation at 25°C. The required primers are listed in Data S7.

### 4.12 BiFC Assay

The CDS sequences of *FveWRKY50*, *FveSnRK2.1* and *FveJAZ5* were constructed into pSPYCE and pSPYNE vectors, respectively, and transferred into Agrobacterium EHA105. Activated Agrobacterium cultures were collected, re‐suspended in infiltration buffer (10‐mM MES pH 5.6, 10‐mM MgCl_2_ and 200‐μM acetosyringone), and incubated at 28°C for 2 h. Each pair was mixed 1:1 and injected into the leaves of *N. benthamiana* at 4–6 weeks of age, and the fluorescence was observed by confocal microscopy 2–3 days later, as described by previous studies (Schütze et al. 2009). The primers used are listed in Data S7.

### 4.13 Recombinant Protein Production and Purification

The CDS sequences of *FveWRKY50* (*FvH4_6g53770*), *FveSnRK2.1* (*FvH4_1g20310*), *FveJAZ5* (*FvH4_6g35140*) and *FveMYB108* (*FvH4_5g11930*) were cloned into pET30a, pGEX4T1 and pCold‐TF (with *FveJAZ5* or *FveMYB108*) vectors, respectively, and transferred into *Escherichia coli* BL21(DE3) cells. The S88A mutations were introduced into FveWRKY50 (FveWRKY50^S88A^) by site‐directed mutagenesis, then transferred into *Escherichia coli* BL21(DE3) cells. Prokaryotic recombinant proteins induced by IPTG were purified according to instructions using Ni‐NTA agarose (Novagen), Glutathione Sepharose beads (GE Healthcare) and BeyoMag Anti‐HA Magnetic Beads (Beyotime, China).

### 4.14 Pull‐Down Assay

Recombinant GST‐FveSnRK2.1, His‐FveWRKY50, HA‐FveJAZ5 and glutathione‐S‐transferase (GST) were purified using glutathione Sepharose beads (GE Healthcare, Chicago, IL, USA), Ni‐NTA agarose (Novagen, Madison, WI, USA) and BeyoMag Anti‐HA Magnetic Beads (Beyotime, China), as per the manufacturer's manuals. His‐FveWRKY50 bound to Ni‐NTA agarose was incubated with GST, GST‐FveSnRK2.1 and HA‐FveJAZ5 at 4°C for 2 h. The beads were washed sequentially with 20, 40 and 60 mM imidazole. The protein was then eluted from the beads using 250 mM imidazole and immunoblotted with Anti‐GST antibody. Similarly, the pull‐down MS assay was conducted by co‐incubating His‐FveWRKY50 with Ni‐NTA agarose beads and total protein extracts from strawberry fruits, followed by imidazole washing and subsequent mass spectrometric analysis.

### 4.15 Plant Protein Extraction and Western Blotting

Add protein extraction buffer (phosphate buffers pH 7.8, 1 mM EDTA, 10% (v/v) glycerol, 0.5% (v/v) Triton X‐100, 1 mM DTT, 1 mM benzoyl sulfonyl fluoride, 1 × protease inhibitor mixture and 1 × phosphatase inhibitor mixture) to strawberry fruits ground into powder with liquid nitrogen and mix well. Samples were placed on ice for 30 min and then centrifuged at 13000 ***g*** for 10 min at 4°C. Proteins in the supernatant were separated using 10% sodium dodecyl sulfate‐polyacrylamide gel electrophoresis (SDS‐PAGE). The antibodies anti‐His (1:1000, CWBIO), anti‐GST (1:1000, CWBIO) and anti‐β‐actin (1:1000, CWBIO) were used for western blotting.

### 4.16 Cell‐Free Degradation Assay

Cell‐free degradation assays were conducted as previously described (Li et al. 2017; Mao et al. 2022). Total fruit proteins were extracted from WT and *FveSnRK2.1*‐overexpressing (OE) plants and incubated with His‐FveWRKY50 or HA‐FveJAZ5 recombinant proteins in buffer (50 mM Tris‐MES pH 8.0, 500 mM sucrose, 1 mM MgCl_2_, 10 mM EDTA and 5 mM DTT). Each mixture was then equally divided into two parts: one supplemented with 50 μM MG132 to inhibit proteasome‐mediated protein degradation and the other treated with DMSO as a control. The samples were incubated at 37°C, and 30 μL aliquots were collected at 0, 30, 60 and 90 min. The levels of His‐FveWRKY50 or HA‐FveJAZ5 proteins were detected by western blotting using anti‐His or anti‐HA antibodies.

### 4.17 Electrophoretic Mobility Shift Assay (EMSA)

His‐FveWRKY50, His‐FveWRKY50^S88A^ and His‐FveMYB108 proteins, as well as biotin‐labelled and unlabeled probes (Sangon Biotech, China), were prepared for the assay. The incubation of probes with proteins, electrophoresis, membrane transfer, cross‐linking and chemiluminescence detection was sequentially performed according to the instructions provided in the Chemiluminescent EMSA Kit (Beyotime, China). The sequences of the probes used in the assay are provided in Data S7.

### 4.18 Phosphorylation Assay

#### 4.18.1 In Vitro Kinase Activity Assay

Kinase activity was assessed using a radiolabeling approach as previously described (Li et al. 2017; Mao et al. 2022). Briefly, His‐FveWRKY50 and GST‐FveSnRK2.1 were combined in a kinase reaction buffer (20 mM Tris–HCl pH 7.5, 10 mM MgCl_2_, 1 mM DTT, 25 μM ATP and 1 μCi [γ‐^32^P] ATP) and incubated at 30°C for 30 min. The reaction was terminated by adding SDS loading buffer, followed by separation of proteins via 10% SDS–PAGE. The gel was dried, exposed to a phosphor imager screen at room temperature for 12–24 h and visualised using a Typhoon 9410 imager.

#### 4.18.2 Phos‐Tag Phosphorylation Assay

Phosphorylation status was validated using Phos‐tag technology (Li et al. 2023). His‐FveWRKY50 or His‐FveWRKY50^S88A^ was individually mixed with GST‐FveSnRK2.1 in a kinase reaction buffer (25 mM Tris–HCl pH 7.5, 10 mM MgCl₂, 1 mM DTT, 50 μM ATP) and incubated at 25°C for 30 min. Fast alkaline phosphatase (Thermo Fisher, USA) was added to the reaction mixture, which was then incubated at 30°C for 30 min to dephosphorylate proteins. Phosphorylated proteins were captured using Phos‐tag Acrylamide (Wako, Japan) according to the manufacturer's protocol. Proteins were separated by SDS–PAGE and detected via immunoblotting with anti‐His (1:1000, CWBIO) or anti‐GST (1:1000, CWBIO) antibodies. To confirm FveSnRK2.1 phosphorylation in planta, total protein extracts from fruit tissues were similarly analysed by immunoblotting with an anti‐FveSnRK2.1 antibody. The FveSnRK2.1 antibody was custom‐produced by Abmart (Shanghai, China). Based on the FveSnRK2.1 protein sequence, specific antigens were designed and synthesised for rabbit immunisation, followed by antibody purification. GST‐tagged FveSnRK2.1 protein was separated by SDS–PAGE and subjected to immunoblotting using both FveSnRK2.1‐specific antibody and anti‐GST antibody, which detected bands of identical molecular weight (Figure S5C).

### 4.19 Phosphorylation Site Identification

His‐FveWRKY50 and GST‐FveSnRK2.1 were incubated in the reaction buffer (25 mM Tris–HCl, pH 7.5, 10 mM MgCl₂, 1 mM DTT and 50 μM ATP) at 25°C for 30 min. Following the reaction, SDS loading buffer was added, and proteins were separated by SDS‐PAGE. The gel was subsequently stained with Coomassie Brilliant Blue (CBB). The gel band corresponding to His‐FveWRKY50 was excised, subjected to tryptic digestion and analysed using a nanoLC‐LTQ‐Orbitrap XL mass spectrometer (Thermo, San Jose, CA, USA).

### 4.20 Statistical Analysis

Data were analysed using two‐tailed Student's *t*‐tests for comparisons between two independent groups (*p < 0.05, **p < 0.01). For multiple group comparisons, one‐way analysis of variance (ANOVA) was performed.

## Author Contributions

Chuang Liu conducted experiments and data analysis. Qian Li and Bingbing Li supervised the project and wrote the manuscript. Yating Chen assisted in obtaining the transgenic materials. Zhen Liu, Xia Li, Ronghui Sun, Peijie Li, Qianqian Feng, Yuanhua Wang and Jie Ren contributed to the experimental work. All authors participated in result discussion and manuscript revision.

## Conflicts of Interest

The authors declare no conflicts of interest.

## Supporting information


**Figure S1:** Identification and genetic transformation of *FveWRKY50*. (A) Number of members from different transcription factor family in *C. gloeosporioides*‐infected strawberry seedlings at 2 dpi, as determined by RNA‐Seq analysis. (B) Expression levels of *FveWRKY50* in various transgenic lines, as measured by qRT‐PCR. Values are means ±SEM of three biological replicates. Statistical significance was determined by Student's *t* test (*p < 0.05, **p < 0.01). (C) Detection of eGFP fluorescence in WT and *FveWRKY50*‐OE fruit. Scale bars, 1 cm. (D) CRISRP/Cas9‐mediated editing patterns in *FveWRKY50*‐CR lines. (E) Phenotypic characterisation and quantification of petiole mortality rates in diploid ‘di Bosco’ following crown infection at different dpi. Values are means ±SEM of three biological replicates. Statistical significance was determined by Student's *t* test (*p < 0.05, **p < 0.01). Scale bars, 2 cm. (F) Growth Phenotypes of WT and *FveWRKY50*‐CR lines. Scale bars, 4 cm.
**Figure S2:** *FveMYB108* was induced upon anthracnose infection and increased *FveWRKY50* expression. (A) The induction expression of *FveMYB108* after anthracnose infection was identified by qRT‐PCR. (B, C) Transient overexpression of *FveMYB108* in octoploid strawberry fruits (B) and determination of the phenotype and lesion area (C). CK, transient expression of empty pH7WG2D vector in octoploid ‘Benihoppe’ fruits as control. (D) The expression of *FveWRKY50*, *FveAOS2* and *FveAOC3* were detected by using qRT‐PCR. Values are means ±SEM of three biological replicates. Statistical significance was determined by Student's *t* test (*p < 0.05, **p < 0.01). E. EMSA was used to identified whether FveMYB108 binds the *FveWRKY50* promoter. *FveWRKY50* promoter probes (P1‐P8) containing candidate MYB binding sties (MBS) were used. Scale bars, 1 cm.
**Figure S3:** Anthracnose increased the content of MeJA in diploid ‘di Bosco’ and octoploid ‘Benihoppe’. (A) The contents of JAs and SAs in WT and *FveWRKY50*‐OE strawberry leaves. (B) MeJA content in different organs of diploid ‘di Bosco’ and octoploid ‘Benihoppe’ strawberries at 3 dpi with *C. gloeosporioides*. S, seedlings; ML, mature leaves; C, crown; F, fruits. Values are means ±SEM of three biological replicates. Statistical significance was determined by Student's *t* test (*p < 0.05, **p < 0.01). (C) Phenotypic characterisation and quantification of lesion areas in *C. gloeosporioides*‐infected ‘di Bosco’ leaves pre‐treated with DIECA (5 and 50 μM, 24 h prior to anthracnose infection) at 3 dpi, 5 dpi and 8 dpi. Scale bars, 1 cm. Values are means ±SEM of three biological replicates. Statistical significance was determined by Student's *t* test (*p < 0.05, **p < 0.01). (D) MeJA content in ‘di Bosco’ leaves treated with 5 and 50 μM DIECA at 24 h. Values are means ±SEM of three biological replicates. Statistical significance was determined by Student's *t* test (*p < 0.05, **p < 0.01).
**Figure S4:** Response patterns of JA biosynthesis genes to anthracnose infection across different strawberry organs. (A–D) Expression profiles of *AOS* and *AOC* genes in (A) seedlings, (B) leaves, (C) crowns and (D) fruits of diploid ‘di Bosco’ and octoploid ‘Benihoppe’ strawberries at 3 dpi with *C. gloeosporioides*, as measured by qRT‐PCR. S, seedlings; ML, mature leaves; C, crown; F, fruits. Values are means ±SEM of three biological replicates. Statistical significance was determined by Student's *t* test (*p < 0.05, **p < 0.01). (E) Expression changes of key JA biosynthesis genes in *C. gloeosporioides*‐infected ‘di Bosco’ seedlings at 2 and 3 dpi, as determined by RNA‐Seq analysis.
**Figure S5:** *FveAOS2* and *FveAOC3* are key marker genes for anthracnose‐induced JA biosynthesis. (A) Time course of MeJA content changes and *FveAOS2*, *FveAOC3* expression in ‘di Bosco’ leaves following anthracnose infection. Values are means ±SEM of three biological replicates. Statistical significance was determined by Student's *t* test (*p < 0.05, **p < 0.01). (B) Expression levels of *FveAOS2* and *FveAOC3* in ‘di Bosco’ leaves treated with DIECA at 24 h, as measured by qRT‐PCR. Values are means ±SEM of three biological replicates. Statistical significance was determined by Student's *t* test (*p < 0.05, **p < 0.01). (C) Transient overexpression of *FveAOC3* increased susceptibility to anthracnose infection in ‘Benihoppe’ fruits. eGFP fluorescence was used to assess transformation efficiency, and *FveAOC3* expression levels were determined by qRT‐PCR. Scale bars, 1 cm. Values are means ±SEM of three biological replicates. Statistical significance was determined by Student's *t* test (*p < 0.05, **p < 0.01). (D) Transient overexpression of *FveAOC3* enhanced MeJA accumulation in ‘Benihoppe’ strawberry fruits. Values are means ±SEM of three biological replicates. Statistical significance was determined by Student's *t* test (*p < 0.05, **p < 0.01).
**Figure S6:** Identification of FveSnRK2.1 and generation of its overexpressing transgenic strawberry plants. (A) Mass spectrometry analysis identified FveSnRK2.1 as an interacting partner of FveWRKY50 in strawberry fruit. The binding peptide of FveSnRK2.1 was indicated in the image. (B) Mass spectrometry analysis identifying Ser88 as the phosphorylation site of His‐FveWRKY50 by GST‐FveSnRK2.1. (C) eGFP fluorescence intensity and *FveSnRK2.1* expression levels in various *FveSnRK2.1*‐OE transgenic lines; *FveSnRK2.1* expression levels were determined by qRT‐PCR. Values are means ±SEM of three biological replicates. Statistical significance was determined by Student's *t* test (*p < 0.05, **p < 0.01). Scale bars, 1 cm. (D) Validation of anti‐FveSnRK2.1 antibody by western blot using GST‐tagged recombinant protein GST‐FveSnRK2.1 (purified and used as a positive control).
**Figure S7:** Phenotypic analysis of *FveMAPK3* transgenics plants. (A) The plant growth phenotypes of WT, *FveMAPK3*‐OE and *FveMAPK3*‐CR seedlings. Scale bar, 1 cm. (B, C) Resistance phenotypic analysis (B) and lesion area quantification (C) in detached leaves (droplet‐inoculated, 5 dpi) of WT and *FveMAPK3*‐ OE strawberry plants. Values are means ±SEM of three biological replicates (10 samples/replicate). Statistical significance was determined by Student's *t* test (*p < 0.05, **p < 0.01). Scale bar, 1 cm.
**Figure S8:** MeJA treatment enhances susceptibility to anthracnose infection in diploid ‘di Bosco’ and octoploid ‘Benihoppe’ strawberries. (A–D) MeJA treatment at different concentrations increased susceptibility to anthracnose infection in detached leaves (A), attached leaves (B), crown‐infected ‘di Bosco’ plants (C), and attached ‘Benihoppe’ leaves (D). Values are means ±SEM of three biological replicates. Statistical significance was determined by Student's *t* test (*p < 0.05, **p < 0.01). (A) Scale bars, 1 cm; (B–D) Scale bars, 2 cm. (E) Exogenous MeJA treatment increased the accumulation of MeJA in attached ‘di Bosco’ leaves at 24 h post‐treatment. Values are means ±SEM of three biological replicates. Statistical significance was determined by Student's *t* test (*p < 0.05, **p < 0.01). (F) Expression changes of *FveAOS2* and *FveAOC3* in attached ‘di Bosco’ leaves under 20 μM MeJA treatment for 24 h, as determined by qRT‐PCR. Values are means ±SEM of three biological replicates. Statistical significance was determined by Student's *t* test (*p < 0.05, **p < 0.01).
**Figure S9:** The identification and genetic transformation of *FveJAZ5*. (A) Basal expression levels of *FveJAZ* genes in ‘di Bosco’ leaves. Values are means ±SEM of three biological replicates. Different lowercase letters indicate significant differences (one‐way ANOVA, Tukey's test). (B) Expression changes of *FveJAZ* genes in *C. gloeosporioides*‐infected ‘di Bosco’ leaves at 24 hpi. Values are means ±SEM of three biological replicates. Statistical significance was determined by Student's *t* test (*p < 0.05, **p < 0.01). (C) Expression levels of *FveJAZ5* in *FveJAZ5*‐OE ‘di Bosco’ plants were determined by qRT‐PCR. Values are means ±SEM of three biological replicates. Statistical significance was determined by Student's *t* test (*p < 0.05, **p < 0.01). (D) CRISRP/Cas9‐mediated editing patterns in *FveWRKY50*‐CR lines. (E) qRT‐PCR analysis validating the expression levels of *FveSnRK2.1*, *FveWRKY50* and *FveJAZ5* were upregulated in transient overexpression fruits. Values are means ±SEM of three biological replicates. Statistical significance was determined by Student's *t* test (*p < 0.05, **p < 0.01). (F) Growth phenotypes of WT and *FveJAZ5*‐OE lines. Scale bars, 2 cm.

**Data S1:** pbi70492‐sup‐0002‐DataS1‐S7.xlsx.

## Data Availability

The data that supports the findings of this study is available in Figures S1–S9 and Data S1–S7 of this article.
