# Supplementary material for: Jasmonate Modulates Strawberry Susceptibility to Anthracnose by Activating SnRK2.1 to Regulate the WRKY50‐JAZ5 Module
Source: Plant Biotechnol J. 2025 Dec 12;24(4):2350–71. doi: 10.1111/pbi.70492 (PMC13140450; doi:10.1111/pbi.70492)
Supplement: Supplementary file 1 — Figure S1: Identification and genetic transformation of FveWRKY50. (A) Number of members from different transcription factor family in C. gloeosporioides‐infected strawberry seedlings at 2 dpi, as determined by RNA‐Seq analysis. (B) Expression levels of FveWRKY50 in various transgenic lines, as measured by qRT‐PCR. Values are means ±SEM of three biological replicates. Statistical significance was determined by Student's t test (*p < 0.05, **p < 0.01). (C) Detection of eGFP fluorescence in WT and FveWRKY50‐OE fruit. Scale bars, 1 cm. (D) CRISRP/Cas9‐mediated editing patterns in FveWRKY50‐CR lines. (E) Phenotypic characterisation and quantification of petiole mortality rates in diploid ‘di Bosco’ following crown infection at different dpi. Values are means ±SEM of three biological replicates. Statistical significance was determined by Student's t test (*p < 0.05, **p < 0.01). Scale bars, 2 cm. (F) Growth Phenotypes of WT and FveWRKY50‐CR lines. Scale bars, 4 cm. Figure S2: FveMYB108 was induced upon anthracnose infection and increased FveWRKY50 expression. (A) The induction expression of FveMYB108 after anthracnose infection was identified by qRT‐PCR. (B, C) Transient overexpression of FveMYB108 in octoploid strawberry fruits (B) and determination of the phenotype and lesion area (C). CK, transient expression of empty pH7WG2D vector in octoploid ‘Benihoppe’ fruits as control. (D) The expression of FveWRKY50, FveAOS2 and FveAOC3 were detected by using qRT‐PCR. Values are means ±SEM of three biological replicates. Statistical significance was determined by Student's t test (*p < 0.05, **p < 0.01). E. EMSA was used to identified whether FveMYB108 binds the FveWRKY50 promoter. FveWRKY50 promoter probes (P1‐P8) containing candidate MYB binding sties (MBS) were used. Scale bars, 1 cm. Figure S3: Anthracnose increased the content of MeJA in diploid ‘di Bosco’ and octoploid ‘Benihoppe’. (A) The contents of JAs and SAs in WT and FveWRKY50‐OE strawberry leaves. (B) MeJA content [file PBI-24-2350-s002.zip › Figure Captions.docx]

**Figure** **S1.** Identification and genetic transformation of *FveWRKY50*. (A) Number of members from different transcription factor family in *C. gloeosporioides*-infected strawberry seedlings at 2 dpi, as determined by RNA-Seq analysis. (B) Expression levels of *FveWRKY50* in various transgenic lines, as measured by qRT-PCR. Values are means ±SEM of three biological replicates. Statistical significance was determined by Student's *t* test (**p* < 0.05, ***p* < 0.01). (C) Detection of eGFP fluorescence in WT and *FveWRKY50*-OE fruit. Scale bars, 1 cm. (D) CRISRP/Cas9-mediated editing patterns in *FveWRKY50*-CR lines. (E) Phenotypic characterisation and quantification of petiole mortality rates in diploid ‘di Bosco’ following crown infection at different dpi. Values are means ±SEM of three biological replicates. Statistical significance was determined by Student's *t* test (**p* < 0.05, ***p* < 0.01). Scale bars, 2 cm. (F) Growth Phenotypes of WT and *FveWRKY50*-CR lines. Scale bars, 4 cm.

**Figure** **S2.** *FveMYB108* was induced upon anthracnose infection and increased *FveWRKY50* expression. (A) The induction expression of *FveMYB108* after anthracnose infection was identified by qRT-PCR. (B, C) Transient overexpression of *FveMYB108* in octoploid strawberry fruits (B) and determination of the phenotype and lesion area (C). CK, transient expression of empty pH7WG2D vector in octoploid ‘Benihoppe’ fruits as control. (D) The expression of *FveWRKY50*, *FveAOS2* and *FveAOC3* were detected by using qRT-PCR. Values are means ±SEM of three biological replicates. Statistical significance was determined by Student's *t* test (**p* < 0.05, ***p* < 0.01). E. EMSA was used to identified whether FveMYB108 binds the *FveWRKY50* promoter. *FveWRKY50* promoter probes (P1-P8) containing candidate MYB binding sties (MBS) were used. Scale bars, 1 cm.

**Figure** **S3.** Anthracnose increased the content of MeJA in diploid ‘di Bosco’ and octoploid ‘Benihoppe’. (A) The contents of JAs and SAs in WT and *FveWRKY50*-OE strawberry leaves. (B) MeJA content in different organs of diploid ‘di Bosco’ and octoploid ‘Benihoppe’ strawberries at 3 dpi with *C. gloeosporioides*. S, seedlings; ML, mature leaves; C, crown; F, fruits. Values are means ±SEM of three biological replicates. Statistical significance was determined by Student's *t* test (**p* < 0.05, ***p* < 0.01). (C) Phenotypic characterisation and quantification of lesion areas in *C. gloeosporioides*-infected ‘di Bosco’ leaves pre-treated with DIECA (5 and 50 μM, 24 h prior to anthracnose infection) at 3 dpi, 5 dpi and 8 dpi. Scale bars, 1 cm. Values are means ±SEM of three biological replicates. Statistical significance was determined by Student's *t* test (**p* < 0.05, ***p* < 0.01). (D) MeJA content in ‘di Bosco’ leaves treated with 5 and 50 μM DIECA at 24 h. Values are means ±SEM of three biological replicates. Statistical significance was determined by Student's *t* test (**p* < 0.05, ***p* < 0.01).

**Figure** **S4.** Response patterns of JA biosynthesis genes to anthracnose infection across different strawberry organs. (A–D) Expression profiles of *AOS* and *AOC* genes in (A) seedlings, (B) leaves, (C) crowns and (D) fruits of diploid ‘di Bosco’ and octoploid ‘Benihoppe’ strawberries at 3 dpi with *C. gloeosporioides*, as measured by qRT-PCR. S, seedlings; ML, mature leaves; C, crown; F, fruits. Values are means ±SEM of three biological replicates. Statistical significance was determined by Student's *t* test (**p* < 0.05, ***p* < 0.01). (E) Expression changes of key JA biosynthesis genes in *C. gloeosporioides*-infected ‘di Bosco’ seedlings at 2 and 3 dpi, as determined by RNA-Seq analysis.

**Figure** **S5.** *FveAOS2* and *FveAOC3* are key marker genes for anthracnose-induced JA biosynthesis. (A) Time course of MeJA content changes and *FveAOS2*, *FveAOC3* expression in ‘di Bosco’ leaves following anthracnose infection. Values are means ±SEM of three biological replicates. Statistical significance was determined by Student's *t* test (**p* < 0.05, ***p* < 0.01). (B) Expression levels of *FveAOS2* and *FveAOC3* in ‘di Bosco’ leaves treated with DIECA at 24 h, as measured by qRT-PCR. Values are means ±SEM of three biological replicates. Statistical significance was determined by Student's *t* test (**p* < 0.05, ***p* < 0.01). (C) Transient overexpression of *FveAOC3* increased susceptibility to anthracnose infection in ‘Benihoppe’ fruits. eGFP fluorescence was used to assess transformation efficiency, and *FveAOC3* expression levels were determined by qRT-PCR. Scale bars, 1 cm. Values are means ±SEM of three biological replicates. Statistical significance was determined by Student's *t* test (**p* < 0.05, ***p* < 0.01). (D) Transient overexpression of *FveAOC3* enhanced MeJA accumulation in ‘Benihoppe’ strawberry fruits. Values are means ±SEM of three biological replicates. Statistical significance was determined by Student's *t* test (**p* < 0.05, ***p* < 0.01).

**Figure** **S6.** Identification of FveSnRK2.1 and generation of its overexpressing transgenic strawberry plants. (A) Mass spectrometry analysis identified FveSnRK2.1 as an interacting partner of FveWRKY50 in strawberry fruit. The binding peptide of FveSnRK2.1 was indicated in the image. (B) Mass spectrometry analysis identifying Ser88 as the phosphorylation site of His-FveWRKY50 by GST-FveSnRK2.1. (C) eGFP fluorescence intensity and *FveSnRK2.1* expression levels in various *FveSnRK2.1*-OE transgenic lines; *FveSnRK2.1* expression levels were determined by qRT-PCR. Values are means ±SEM of three biological replicates. Statistical significance was determined by Student's *t* test (**p* < 0.05, ***p* < 0.01). Scale bars, 1 cm. (D) Validation of anti-FveSnRK2.1 antibody by western blot using GST-tagged recombinant protein GST-FveSnRK2.1 (purified and used as a positive control).

**Figure** **S7.** Phenotypic analysis of *FveMAPK3* transgenics plants. (A) The plant growth phenotypes of WT, *FveMAPK3*-OE and *FveMAPK3*-CR seedlings. Scale bar, 1 cm. (B, C) Resistance phenotypic analysis (B) and lesion area quantification (C) in detached leaves (droplet-inoculated, 5 dpi) of WT and *FveMAPK3*- OE strawberry plants. Values are means ±SEM of three biological replicates (10 samples/replicate). Statistical significance was determined by Student's *t* test (**p* < 0.05, ***p* < 0.01). Scale bar, 1 cm.

**Figure** **S8.** MeJA treatment enhances susceptibility to anthracnose infection in diploid ‘di Bosco’ and octoploid ‘Benihoppe’ strawberries. (A–D) MeJA treatment at different concentrations increased susceptibility to anthracnose infection in detached leaves (A), attached leaves (B), crown-infected ‘di Bosco’ plants (C), and attached ‘Benihoppe’ leaves (D). Values are means ±SEM of three biological replicates. Statistical significance was determined by Student's *t* test (**p* < 0.05, ***p* < 0.01). (A) Scale bars, 1 cm; (B–D) Scale bars, 2 cm. (E) Exogenous MeJA treatment increased the accumulation of MeJA in attached ‘di Bosco’ leaves at 24 h post-treatment. Values are means ±SEM of three biological replicates. Statistical significance was determined by Student's *t* test (**p* < 0.05, ***p* < 0.01). (F) Expression changes of *FveAOS2* and *FveAOC3* in attached ‘di Bosco’ leaves under 20 μM MeJA treatment for 24 h, as determined by qRT-PCR. Values are means ±SEM of three biological replicates. Statistical significance was determined by Student's *t* test (**p* < 0.05, ***p* < 0.01).

**Figure** **S9.** The identification and genetic transformation of *FveJAZ5.* (A) Basal expression levels of *FveJAZ* genes in ‘di Bosco’ leaves. Values are means ±SEM of three biological replicates. Different lowercase letters indicate significant differences (one-way ANOVA, Tukey's test). (B) Expression changes of *FveJAZ* genes in *C. gloeosporioides* -infected ‘di Bosco’ leaves at 24 hpi. Values are means ±SEM of three biological replicates. Statistical significance was determined by Student's *t* test (**p* < 0.05, ***p* < 0.01). (C) Expression levels of *FveJAZ5* in *FveJAZ5*-OE ‘di Bosco’ plants were determined by qRT-PCR. Values are means ±SEM of three biological replicates. Statistical significance was determined by Student's *t* test (**p* < 0.05, ***p* < 0.01). (D) CRISRP/Cas9-mediated editing patterns in *FveWRKY50*-CR lines. (E) qRT-PCR analysis validating the expression levels of *FveSnRK2.1*, *FveWRKY50* and *FveJAZ5* were upregulated in transient overexpression fruits. Values are means ±SEM of three biological replicates. Statistical significance was determined by Student's *t* test (**p* < 0.05, ***p* < 0.01). (F) Growth phenotypes of WT and *FveJAZ5*-OE lines. Scale bars, 2 cm.
