# Supplementary figures and images for: Jasmonate Modulates Strawberry Susceptibility to Anthracnose by Activating SnRK2.1 to Regulate the WRKY50‐JAZ5 Module
Source: Plant Biotechnol J. 2025 Dec 12;24(4):2350–71. doi: 10.1111/pbi.70492 (PMC13140450; doi:10.1111/pbi.70492)

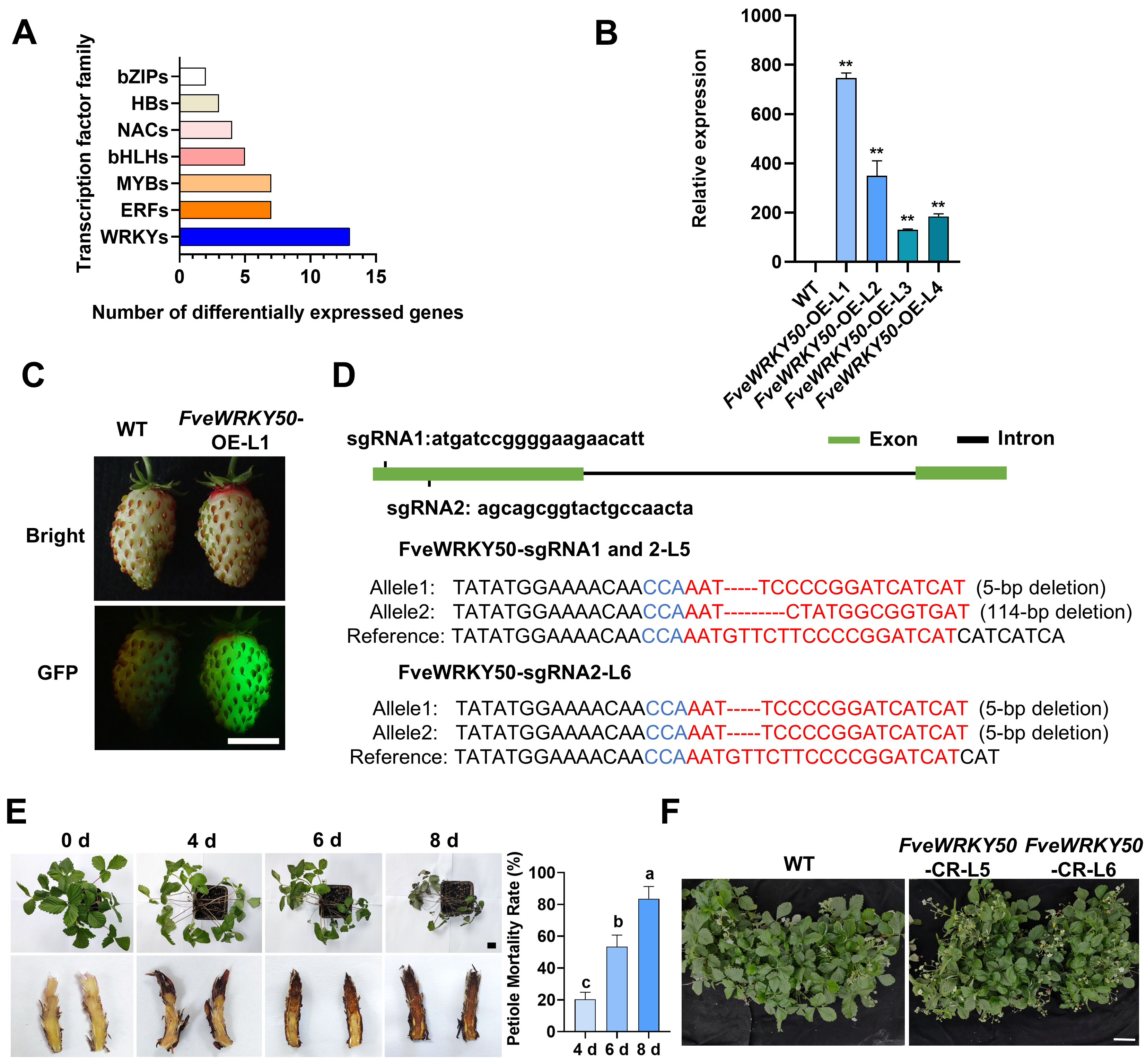

Supplement: Supplementary file 1 — Figure S1: Identification and genetic transformation of FveWRKY50. (A) Number of members from different transcription factor family in C. gloeosporioides‐infected strawberry seedlings at 2 dpi, as determined by RNA‐Seq analysis. (B) Expression levels of FveWRKY50 in various transgenic lines, as measured by qRT‐PCR. Values are means ±SEM of three biological replicates. Statistical significance was determined by Student's t test (*p < 0.05, **p < 0.01). (C) Detection of eGFP fluorescence in WT and FveWRKY50‐OE fruit. Scale bars, 1 cm. (D) CRISRP/Cas9‐mediated editing patterns in FveWRKY50‐CR lines. (E) Phenotypic characterisation and quantification of petiole mortality rates in diploid ‘di Bosco’ following crown infection at different dpi. Values are means ±SEM of three biological replicates. Statistical significance was determined by Student's t test (*p < 0.05, **p < 0.01). Scale bars, 2 cm. (F) Growth Phenotypes of WT and FveWRKY50‐CR lines. Scale bars, 4 cm. Figure S2: FveMYB108 was induced upon anthracnose infection and increased FveWRKY50 expression. (A) The induction expression of FveMYB108 after anthracnose infection was identified by qRT‐PCR. (B, C) Transient overexpression of FveMYB108 in octoploid strawberry fruits (B) and determination of the phenotype and lesion area (C). CK, transient expression of empty pH7WG2D vector in octoploid ‘Benihoppe’ fruits as control. (D) The expression of FveWRKY50, FveAOS2 and FveAOC3 were detected by using qRT‐PCR. Values are means ±SEM of three biological replicates. Statistical significance was determined by Student's t test (*p < 0.05, **p < 0.01). E. EMSA was used to identified whether FveMYB108 binds the FveWRKY50 promoter. FveWRKY50 promoter probes (P1‐P8) containing candidate MYB binding sties (MBS) were used. Scale bars, 1 cm. Figure S3: Anthracnose increased the content of MeJA in diploid ‘di Bosco’ and octoploid ‘Benihoppe’. (A) The contents of JAs and SAs in WT and FveWRKY50‐OE strawberry leaves. (B) MeJA content [file PBI-24-2350-s002.zip › pbi70492-sup-0001-FigureS1@Fig.S1.tif]

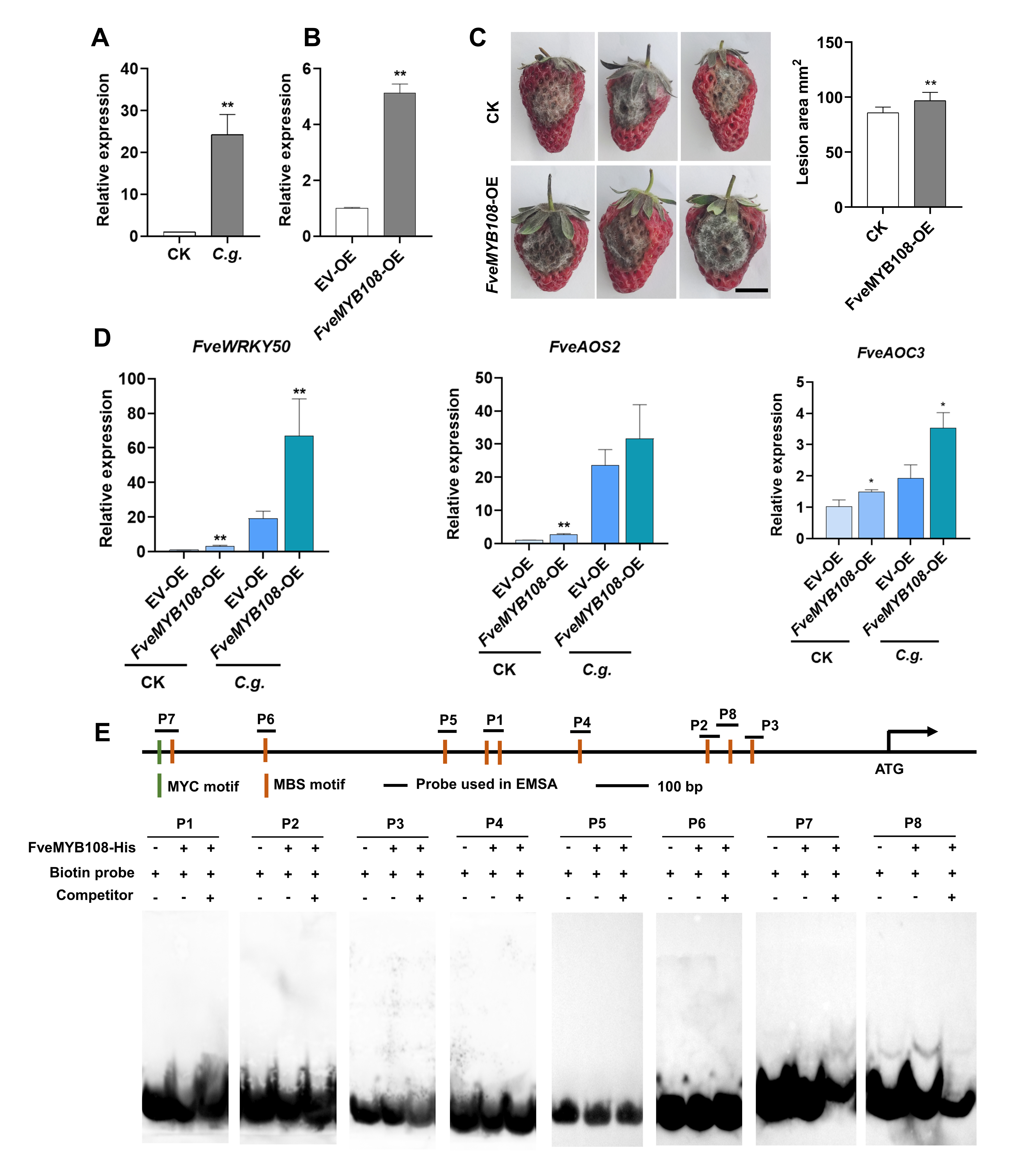

Supplement: Supplementary file 1 — Figure S1: Identification and genetic transformation of FveWRKY50. (A) Number of members from different transcription factor family in C. gloeosporioides‐infected strawberry seedlings at 2 dpi, as determined by RNA‐Seq analysis. (B) Expression levels of FveWRKY50 in various transgenic lines, as measured by qRT‐PCR. Values are means ±SEM of three biological replicates. Statistical significance was determined by Student's t test (*p < 0.05, **p < 0.01). (C) Detection of eGFP fluorescence in WT and FveWRKY50‐OE fruit. Scale bars, 1 cm. (D) CRISRP/Cas9‐mediated editing patterns in FveWRKY50‐CR lines. (E) Phenotypic characterisation and quantification of petiole mortality rates in diploid ‘di Bosco’ following crown infection at different dpi. Values are means ±SEM of three biological replicates. Statistical significance was determined by Student's t test (*p < 0.05, **p < 0.01). Scale bars, 2 cm. (F) Growth Phenotypes of WT and FveWRKY50‐CR lines. Scale bars, 4 cm. Figure S2: FveMYB108 was induced upon anthracnose infection and increased FveWRKY50 expression. (A) The induction expression of FveMYB108 after anthracnose infection was identified by qRT‐PCR. (B, C) Transient overexpression of FveMYB108 in octoploid strawberry fruits (B) and determination of the phenotype and lesion area (C). CK, transient expression of empty pH7WG2D vector in octoploid ‘Benihoppe’ fruits as control. (D) The expression of FveWRKY50, FveAOS2 and FveAOC3 were detected by using qRT‐PCR. Values are means ±SEM of three biological replicates. Statistical significance was determined by Student's t test (*p < 0.05, **p < 0.01). E. EMSA was used to identified whether FveMYB108 binds the FveWRKY50 promoter. FveWRKY50 promoter probes (P1‐P8) containing candidate MYB binding sties (MBS) were used. Scale bars, 1 cm. Figure S3: Anthracnose increased the content of MeJA in diploid ‘di Bosco’ and octoploid ‘Benihoppe’. (A) The contents of JAs and SAs in WT and FveWRKY50‐OE strawberry leaves. (B) MeJA content [file PBI-24-2350-s002.zip › pbi70492-sup-0002-FigureS2@Fig.S2.jpg]

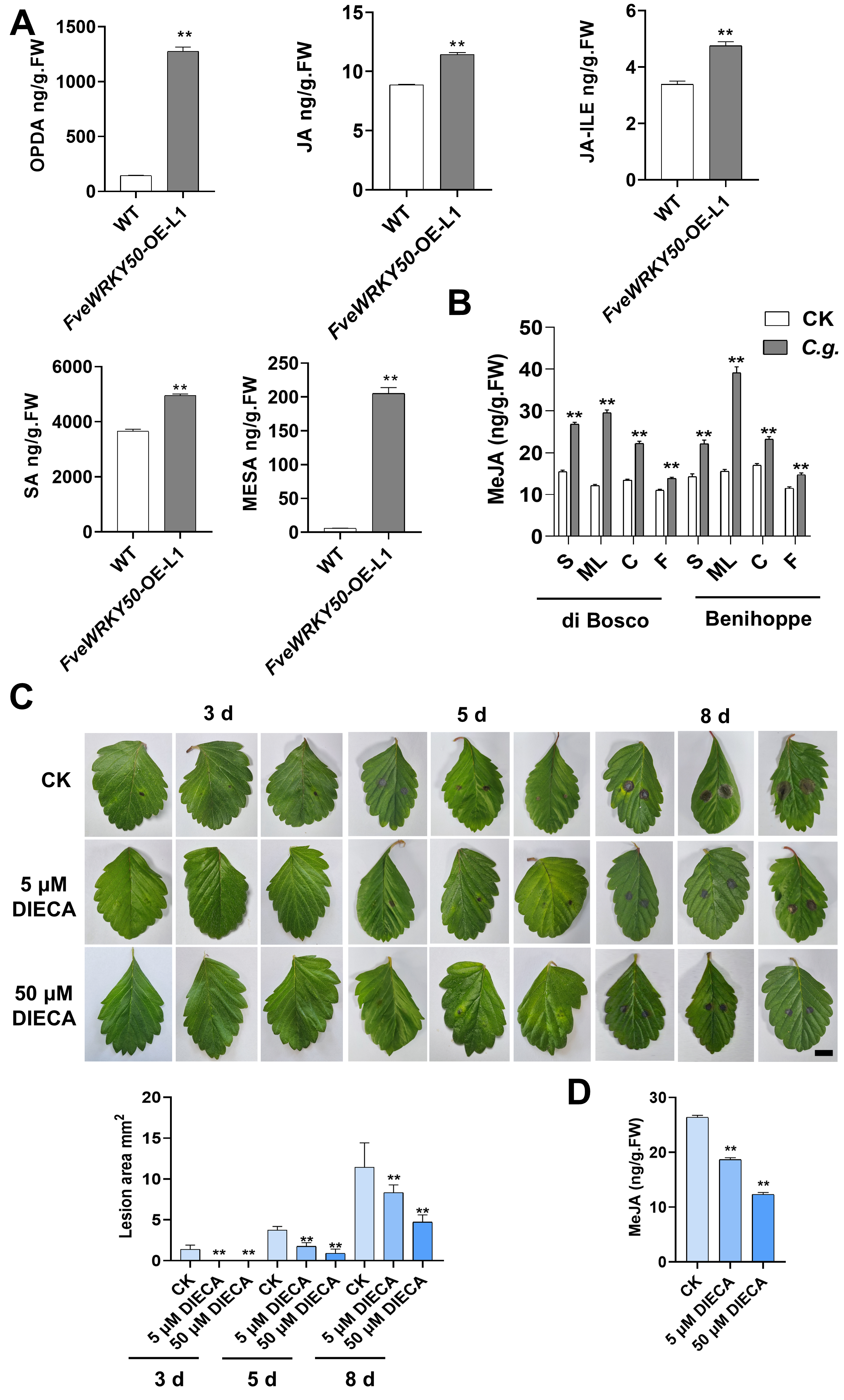

Supplement: Supplementary file 1 — Figure S1: Identification and genetic transformation of FveWRKY50. (A) Number of members from different transcription factor family in C. gloeosporioides‐infected strawberry seedlings at 2 dpi, as determined by RNA‐Seq analysis. (B) Expression levels of FveWRKY50 in various transgenic lines, as measured by qRT‐PCR. Values are means ±SEM of three biological replicates. Statistical significance was determined by Student's t test (*p < 0.05, **p < 0.01). (C) Detection of eGFP fluorescence in WT and FveWRKY50‐OE fruit. Scale bars, 1 cm. (D) CRISRP/Cas9‐mediated editing patterns in FveWRKY50‐CR lines. (E) Phenotypic characterisation and quantification of petiole mortality rates in diploid ‘di Bosco’ following crown infection at different dpi. Values are means ±SEM of three biological replicates. Statistical significance was determined by Student's t test (*p < 0.05, **p < 0.01). Scale bars, 2 cm. (F) Growth Phenotypes of WT and FveWRKY50‐CR lines. Scale bars, 4 cm. Figure S2: FveMYB108 was induced upon anthracnose infection and increased FveWRKY50 expression. (A) The induction expression of FveMYB108 after anthracnose infection was identified by qRT‐PCR. (B, C) Transient overexpression of FveMYB108 in octoploid strawberry fruits (B) and determination of the phenotype and lesion area (C). CK, transient expression of empty pH7WG2D vector in octoploid ‘Benihoppe’ fruits as control. (D) The expression of FveWRKY50, FveAOS2 and FveAOC3 were detected by using qRT‐PCR. Values are means ±SEM of three biological replicates. Statistical significance was determined by Student's t test (*p < 0.05, **p < 0.01). E. EMSA was used to identified whether FveMYB108 binds the FveWRKY50 promoter. FveWRKY50 promoter probes (P1‐P8) containing candidate MYB binding sties (MBS) were used. Scale bars, 1 cm. Figure S3: Anthracnose increased the content of MeJA in diploid ‘di Bosco’ and octoploid ‘Benihoppe’. (A) The contents of JAs and SAs in WT and FveWRKY50‐OE strawberry leaves. (B) MeJA content [file PBI-24-2350-s002.zip › pbi70492-sup-0003-FigureS3@Fig.S3.tif]

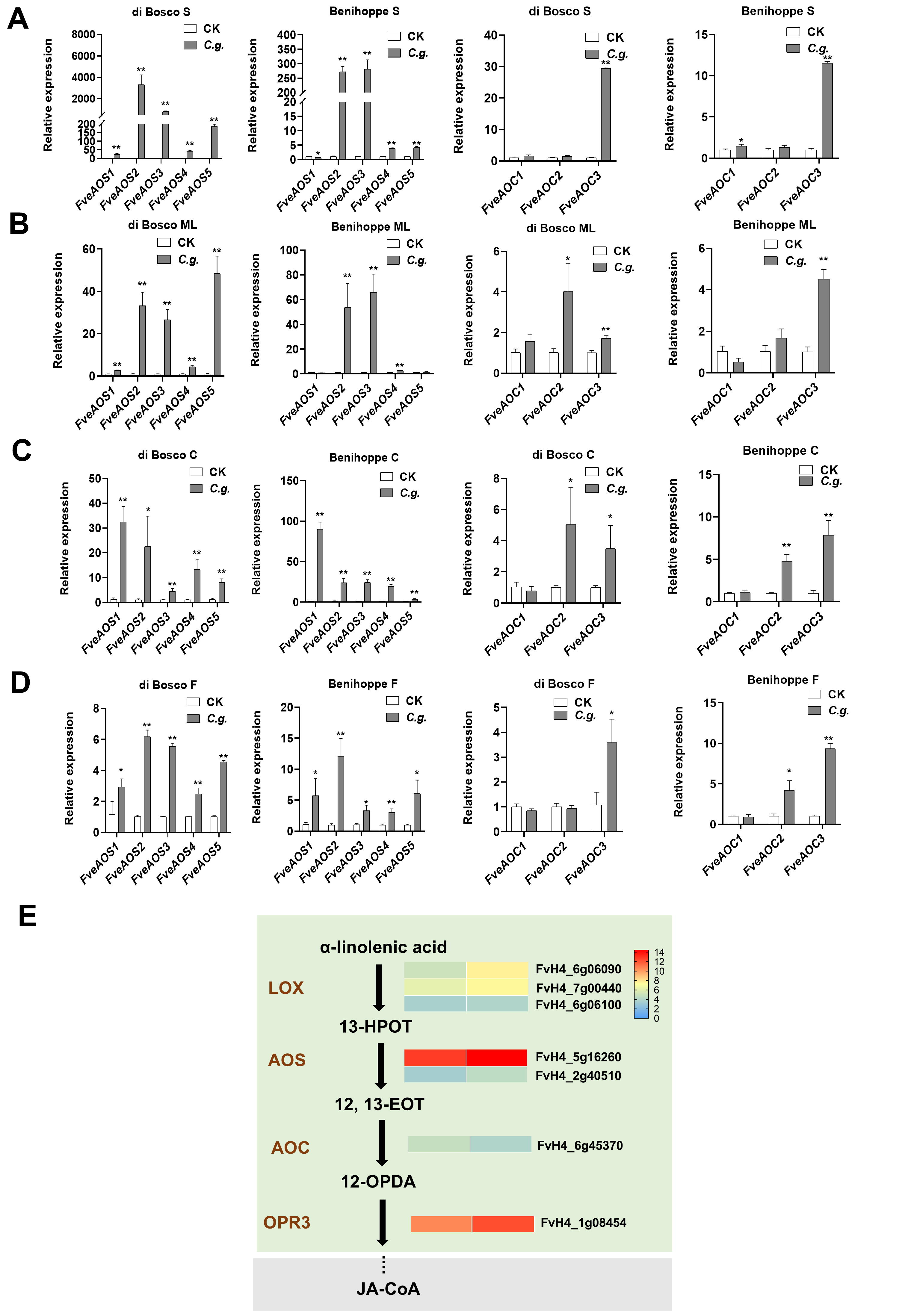

Supplement: Supplementary file 1 — Figure S1: Identification and genetic transformation of FveWRKY50. (A) Number of members from different transcription factor family in C. gloeosporioides‐infected strawberry seedlings at 2 dpi, as determined by RNA‐Seq analysis. (B) Expression levels of FveWRKY50 in various transgenic lines, as measured by qRT‐PCR. Values are means ±SEM of three biological replicates. Statistical significance was determined by Student's t test (*p < 0.05, **p < 0.01). (C) Detection of eGFP fluorescence in WT and FveWRKY50‐OE fruit. Scale bars, 1 cm. (D) CRISRP/Cas9‐mediated editing patterns in FveWRKY50‐CR lines. (E) Phenotypic characterisation and quantification of petiole mortality rates in diploid ‘di Bosco’ following crown infection at different dpi. Values are means ±SEM of three biological replicates. Statistical significance was determined by Student's t test (*p < 0.05, **p < 0.01). Scale bars, 2 cm. (F) Growth Phenotypes of WT and FveWRKY50‐CR lines. Scale bars, 4 cm. Figure S2: FveMYB108 was induced upon anthracnose infection and increased FveWRKY50 expression. (A) The induction expression of FveMYB108 after anthracnose infection was identified by qRT‐PCR. (B, C) Transient overexpression of FveMYB108 in octoploid strawberry fruits (B) and determination of the phenotype and lesion area (C). CK, transient expression of empty pH7WG2D vector in octoploid ‘Benihoppe’ fruits as control. (D) The expression of FveWRKY50, FveAOS2 and FveAOC3 were detected by using qRT‐PCR. Values are means ±SEM of three biological replicates. Statistical significance was determined by Student's t test (*p < 0.05, **p < 0.01). E. EMSA was used to identified whether FveMYB108 binds the FveWRKY50 promoter. FveWRKY50 promoter probes (P1‐P8) containing candidate MYB binding sties (MBS) were used. Scale bars, 1 cm. Figure S3: Anthracnose increased the content of MeJA in diploid ‘di Bosco’ and octoploid ‘Benihoppe’. (A) The contents of JAs and SAs in WT and FveWRKY50‐OE strawberry leaves. (B) MeJA content [file PBI-24-2350-s002.zip › pbi70492-sup-0004-FigureS4@Fig.S4.tif]

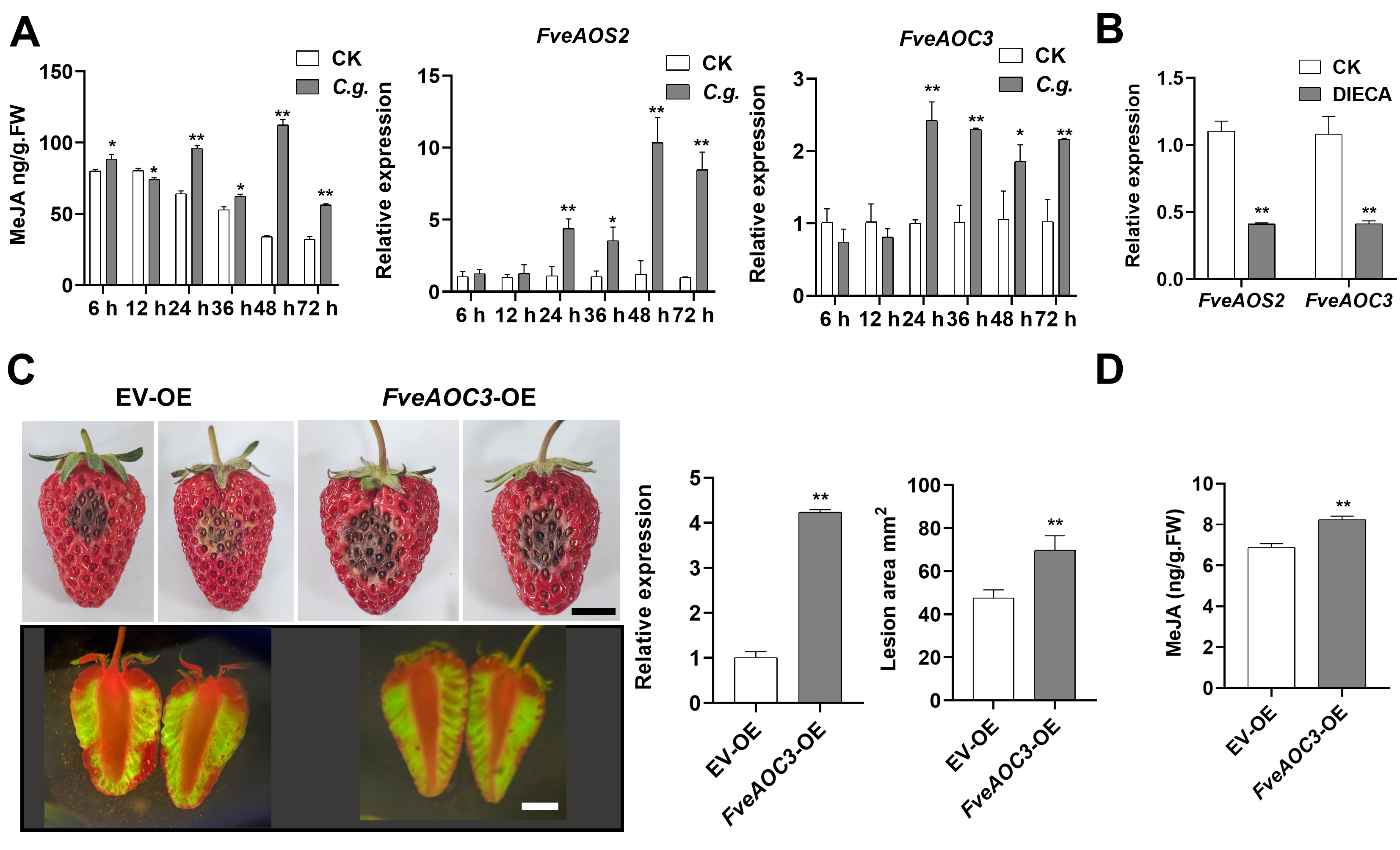

Supplement: Supplementary file 1 — Figure S1: Identification and genetic transformation of FveWRKY50. (A) Number of members from different transcription factor family in C. gloeosporioides‐infected strawberry seedlings at 2 dpi, as determined by RNA‐Seq analysis. (B) Expression levels of FveWRKY50 in various transgenic lines, as measured by qRT‐PCR. Values are means ±SEM of three biological replicates. Statistical significance was determined by Student's t test (*p < 0.05, **p < 0.01). (C) Detection of eGFP fluorescence in WT and FveWRKY50‐OE fruit. Scale bars, 1 cm. (D) CRISRP/Cas9‐mediated editing patterns in FveWRKY50‐CR lines. (E) Phenotypic characterisation and quantification of petiole mortality rates in diploid ‘di Bosco’ following crown infection at different dpi. Values are means ±SEM of three biological replicates. Statistical significance was determined by Student's t test (*p < 0.05, **p < 0.01). Scale bars, 2 cm. (F) Growth Phenotypes of WT and FveWRKY50‐CR lines. Scale bars, 4 cm. Figure S2: FveMYB108 was induced upon anthracnose infection and increased FveWRKY50 expression. (A) The induction expression of FveMYB108 after anthracnose infection was identified by qRT‐PCR. (B, C) Transient overexpression of FveMYB108 in octoploid strawberry fruits (B) and determination of the phenotype and lesion area (C). CK, transient expression of empty pH7WG2D vector in octoploid ‘Benihoppe’ fruits as control. (D) The expression of FveWRKY50, FveAOS2 and FveAOC3 were detected by using qRT‐PCR. Values are means ±SEM of three biological replicates. Statistical significance was determined by Student's t test (*p < 0.05, **p < 0.01). E. EMSA was used to identified whether FveMYB108 binds the FveWRKY50 promoter. FveWRKY50 promoter probes (P1‐P8) containing candidate MYB binding sties (MBS) were used. Scale bars, 1 cm. Figure S3: Anthracnose increased the content of MeJA in diploid ‘di Bosco’ and octoploid ‘Benihoppe’. (A) The contents of JAs and SAs in WT and FveWRKY50‐OE strawberry leaves. (B) MeJA content [file PBI-24-2350-s002.zip › pbi70492-sup-0005-FigureS5@Fig.S5.tif]

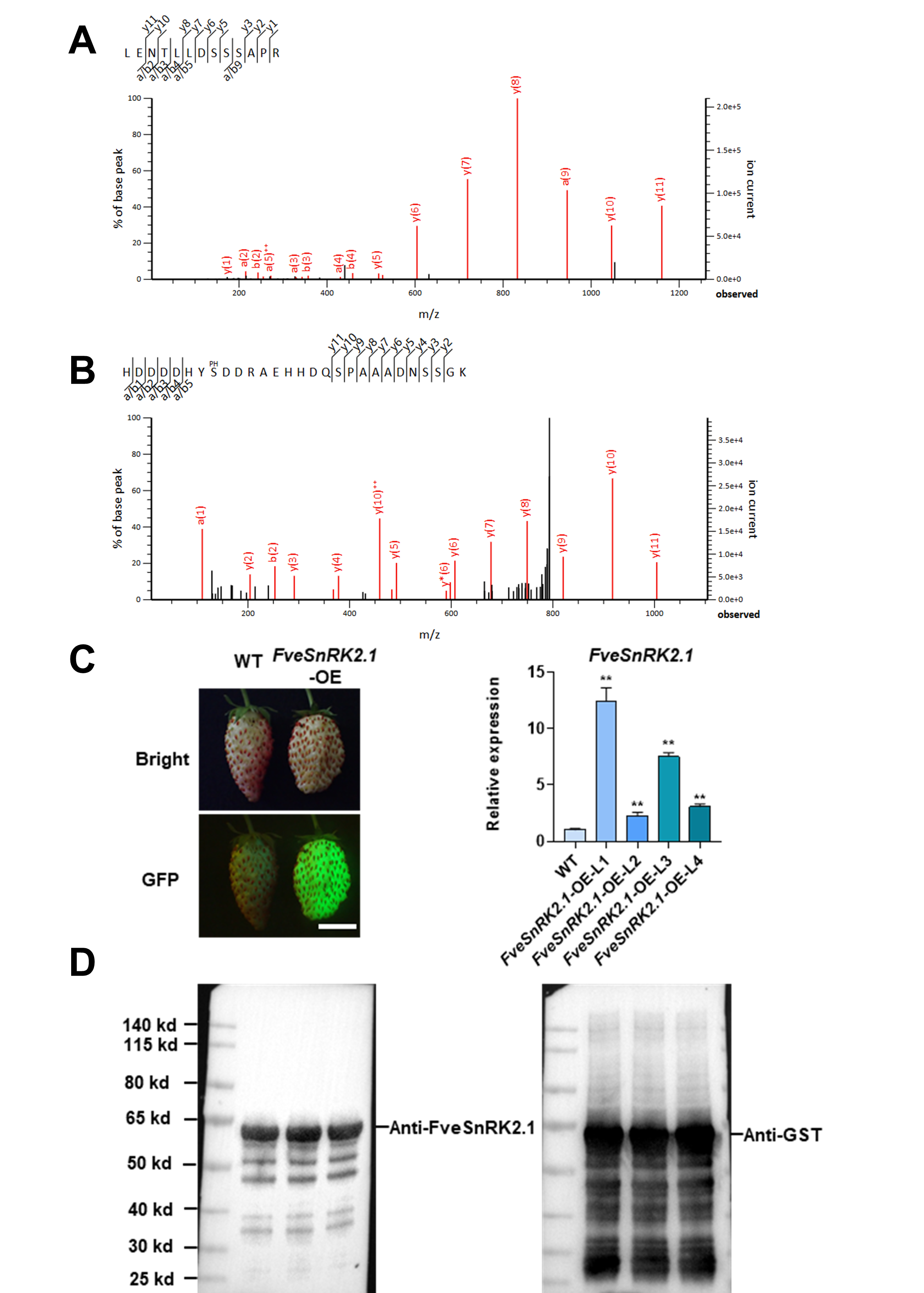

Supplement: Supplementary file 1 — Figure S1: Identification and genetic transformation of FveWRKY50. (A) Number of members from different transcription factor family in C. gloeosporioides‐infected strawberry seedlings at 2 dpi, as determined by RNA‐Seq analysis. (B) Expression levels of FveWRKY50 in various transgenic lines, as measured by qRT‐PCR. Values are means ±SEM of three biological replicates. Statistical significance was determined by Student's t test (*p < 0.05, **p < 0.01). (C) Detection of eGFP fluorescence in WT and FveWRKY50‐OE fruit. Scale bars, 1 cm. (D) CRISRP/Cas9‐mediated editing patterns in FveWRKY50‐CR lines. (E) Phenotypic characterisation and quantification of petiole mortality rates in diploid ‘di Bosco’ following crown infection at different dpi. Values are means ±SEM of three biological replicates. Statistical significance was determined by Student's t test (*p < 0.05, **p < 0.01). Scale bars, 2 cm. (F) Growth Phenotypes of WT and FveWRKY50‐CR lines. Scale bars, 4 cm. Figure S2: FveMYB108 was induced upon anthracnose infection and increased FveWRKY50 expression. (A) The induction expression of FveMYB108 after anthracnose infection was identified by qRT‐PCR. (B, C) Transient overexpression of FveMYB108 in octoploid strawberry fruits (B) and determination of the phenotype and lesion area (C). CK, transient expression of empty pH7WG2D vector in octoploid ‘Benihoppe’ fruits as control. (D) The expression of FveWRKY50, FveAOS2 and FveAOC3 were detected by using qRT‐PCR. Values are means ±SEM of three biological replicates. Statistical significance was determined by Student's t test (*p < 0.05, **p < 0.01). E. EMSA was used to identified whether FveMYB108 binds the FveWRKY50 promoter. FveWRKY50 promoter probes (P1‐P8) containing candidate MYB binding sties (MBS) were used. Scale bars, 1 cm. Figure S3: Anthracnose increased the content of MeJA in diploid ‘di Bosco’ and octoploid ‘Benihoppe’. (A) The contents of JAs and SAs in WT and FveWRKY50‐OE strawberry leaves. (B) MeJA content [file PBI-24-2350-s002.zip › pbi70492-sup-0006-FigureS6@Figure S6.tif]

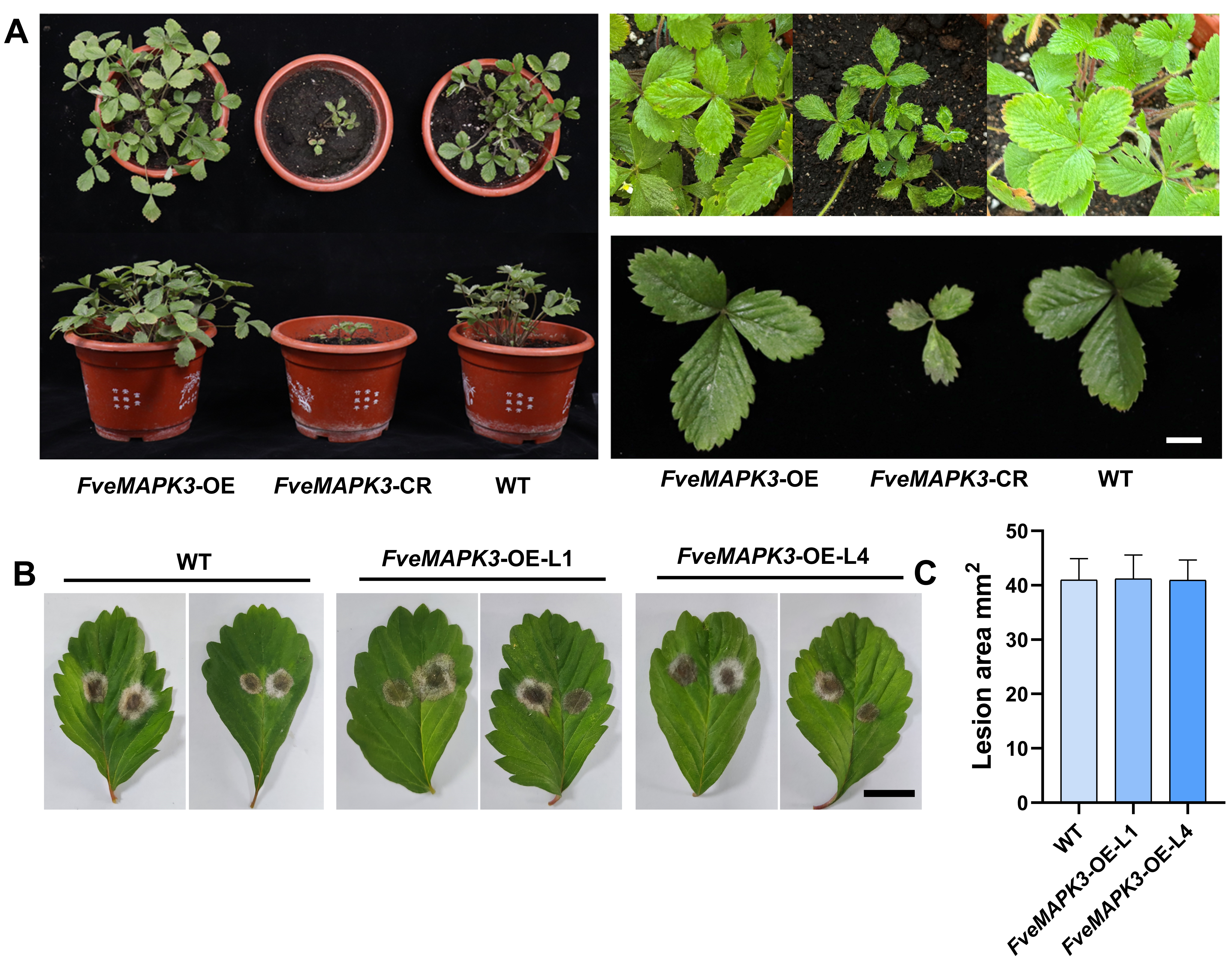

Supplement: Supplementary file 1 — Figure S1: Identification and genetic transformation of FveWRKY50. (A) Number of members from different transcription factor family in C. gloeosporioides‐infected strawberry seedlings at 2 dpi, as determined by RNA‐Seq analysis. (B) Expression levels of FveWRKY50 in various transgenic lines, as measured by qRT‐PCR. Values are means ±SEM of three biological replicates. Statistical significance was determined by Student's t test (*p < 0.05, **p < 0.01). (C) Detection of eGFP fluorescence in WT and FveWRKY50‐OE fruit. Scale bars, 1 cm. (D) CRISRP/Cas9‐mediated editing patterns in FveWRKY50‐CR lines. (E) Phenotypic characterisation and quantification of petiole mortality rates in diploid ‘di Bosco’ following crown infection at different dpi. Values are means ±SEM of three biological replicates. Statistical significance was determined by Student's t test (*p < 0.05, **p < 0.01). Scale bars, 2 cm. (F) Growth Phenotypes of WT and FveWRKY50‐CR lines. Scale bars, 4 cm. Figure S2: FveMYB108 was induced upon anthracnose infection and increased FveWRKY50 expression. (A) The induction expression of FveMYB108 after anthracnose infection was identified by qRT‐PCR. (B, C) Transient overexpression of FveMYB108 in octoploid strawberry fruits (B) and determination of the phenotype and lesion area (C). CK, transient expression of empty pH7WG2D vector in octoploid ‘Benihoppe’ fruits as control. (D) The expression of FveWRKY50, FveAOS2 and FveAOC3 were detected by using qRT‐PCR. Values are means ±SEM of three biological replicates. Statistical significance was determined by Student's t test (*p < 0.05, **p < 0.01). E. EMSA was used to identified whether FveMYB108 binds the FveWRKY50 promoter. FveWRKY50 promoter probes (P1‐P8) containing candidate MYB binding sties (MBS) were used. Scale bars, 1 cm. Figure S3: Anthracnose increased the content of MeJA in diploid ‘di Bosco’ and octoploid ‘Benihoppe’. (A) The contents of JAs and SAs in WT and FveWRKY50‐OE strawberry leaves. (B) MeJA content [file PBI-24-2350-s002.zip › pbi70492-sup-0007-FigureS7@Fig.S7.jpg]

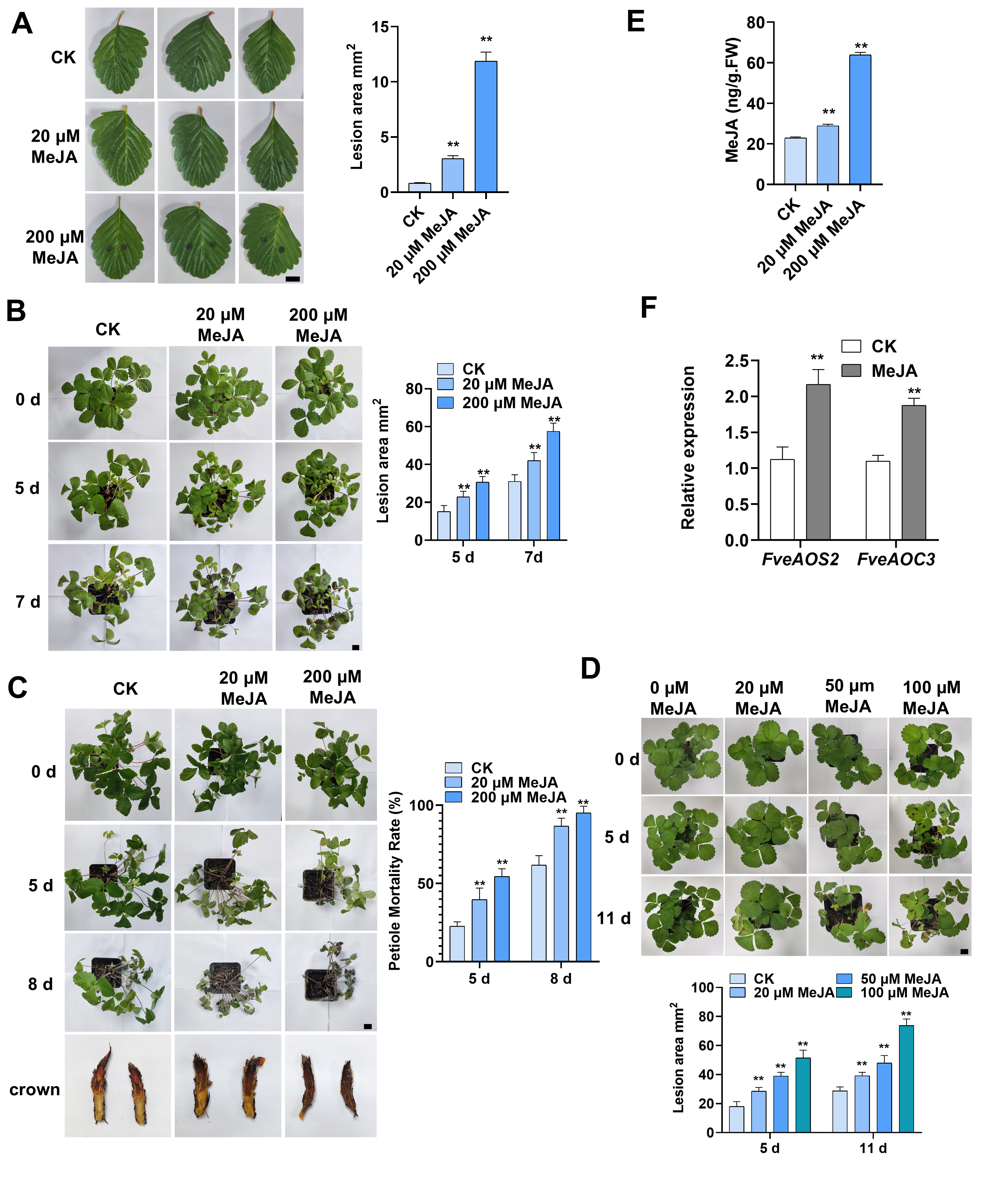

Supplement: Supplementary file 1 — Figure S1: Identification and genetic transformation of FveWRKY50. (A) Number of members from different transcription factor family in C. gloeosporioides‐infected strawberry seedlings at 2 dpi, as determined by RNA‐Seq analysis. (B) Expression levels of FveWRKY50 in various transgenic lines, as measured by qRT‐PCR. Values are means ±SEM of three biological replicates. Statistical significance was determined by Student's t test (*p < 0.05, **p < 0.01). (C) Detection of eGFP fluorescence in WT and FveWRKY50‐OE fruit. Scale bars, 1 cm. (D) CRISRP/Cas9‐mediated editing patterns in FveWRKY50‐CR lines. (E) Phenotypic characterisation and quantification of petiole mortality rates in diploid ‘di Bosco’ following crown infection at different dpi. Values are means ±SEM of three biological replicates. Statistical significance was determined by Student's t test (*p < 0.05, **p < 0.01). Scale bars, 2 cm. (F) Growth Phenotypes of WT and FveWRKY50‐CR lines. Scale bars, 4 cm. Figure S2: FveMYB108 was induced upon anthracnose infection and increased FveWRKY50 expression. (A) The induction expression of FveMYB108 after anthracnose infection was identified by qRT‐PCR. (B, C) Transient overexpression of FveMYB108 in octoploid strawberry fruits (B) and determination of the phenotype and lesion area (C). CK, transient expression of empty pH7WG2D vector in octoploid ‘Benihoppe’ fruits as control. (D) The expression of FveWRKY50, FveAOS2 and FveAOC3 were detected by using qRT‐PCR. Values are means ±SEM of three biological replicates. Statistical significance was determined by Student's t test (*p < 0.05, **p < 0.01). E. EMSA was used to identified whether FveMYB108 binds the FveWRKY50 promoter. FveWRKY50 promoter probes (P1‐P8) containing candidate MYB binding sties (MBS) were used. Scale bars, 1 cm. Figure S3: Anthracnose increased the content of MeJA in diploid ‘di Bosco’ and octoploid ‘Benihoppe’. (A) The contents of JAs and SAs in WT and FveWRKY50‐OE strawberry leaves. (B) MeJA content [file PBI-24-2350-s002.zip › pbi70492-sup-0008-FigureS8@Fig.S8.tif]

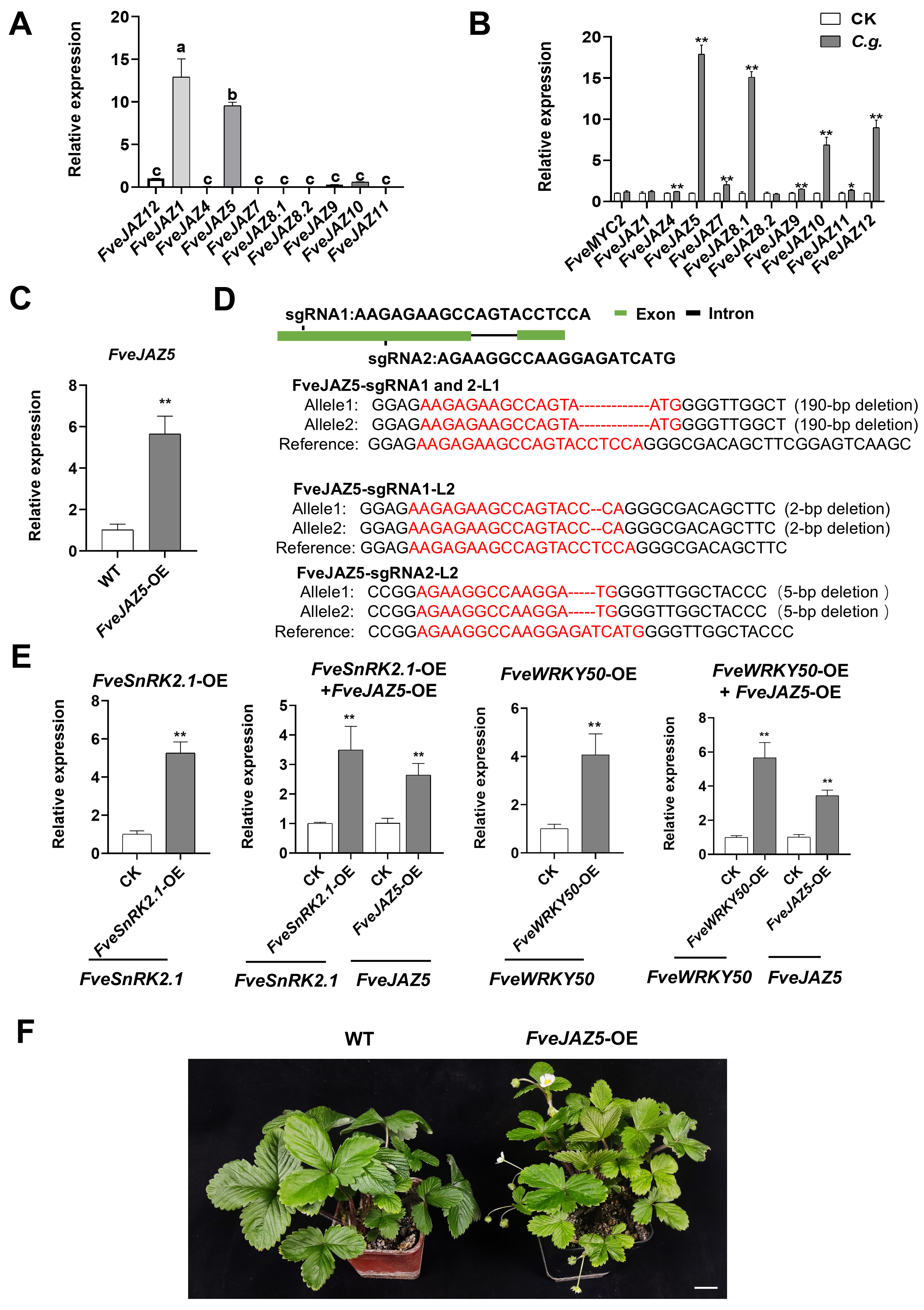

Supplement: Supplementary file 1 — Figure S1: Identification and genetic transformation of FveWRKY50. (A) Number of members from different transcription factor family in C. gloeosporioides‐infected strawberry seedlings at 2 dpi, as determined by RNA‐Seq analysis. (B) Expression levels of FveWRKY50 in various transgenic lines, as measured by qRT‐PCR. Values are means ±SEM of three biological replicates. Statistical significance was determined by Student's t test (*p < 0.05, **p < 0.01). (C) Detection of eGFP fluorescence in WT and FveWRKY50‐OE fruit. Scale bars, 1 cm. (D) CRISRP/Cas9‐mediated editing patterns in FveWRKY50‐CR lines. (E) Phenotypic characterisation and quantification of petiole mortality rates in diploid ‘di Bosco’ following crown infection at different dpi. Values are means ±SEM of three biological replicates. Statistical significance was determined by Student's t test (*p < 0.05, **p < 0.01). Scale bars, 2 cm. (F) Growth Phenotypes of WT and FveWRKY50‐CR lines. Scale bars, 4 cm. Figure S2: FveMYB108 was induced upon anthracnose infection and increased FveWRKY50 expression. (A) The induction expression of FveMYB108 after anthracnose infection was identified by qRT‐PCR. (B, C) Transient overexpression of FveMYB108 in octoploid strawberry fruits (B) and determination of the phenotype and lesion area (C). CK, transient expression of empty pH7WG2D vector in octoploid ‘Benihoppe’ fruits as control. (D) The expression of FveWRKY50, FveAOS2 and FveAOC3 were detected by using qRT‐PCR. Values are means ±SEM of three biological replicates. Statistical significance was determined by Student's t test (*p < 0.05, **p < 0.01). E. EMSA was used to identified whether FveMYB108 binds the FveWRKY50 promoter. FveWRKY50 promoter probes (P1‐P8) containing candidate MYB binding sties (MBS) were used. Scale bars, 1 cm. Figure S3: Anthracnose increased the content of MeJA in diploid ‘di Bosco’ and octoploid ‘Benihoppe’. (A) The contents of JAs and SAs in WT and FveWRKY50‐OE strawberry leaves. (B) MeJA content [file PBI-24-2350-s002.zip › pbi70492-sup-0009-FigureS9@Fig.S9.tif]
